# Mechanistic Insights into the Photochemical Formation of Carbenes from Phenyl-trifluoromethyl Diazirine and Its Diazo Isomer: An MS-CASPT2 Study

**DOI:** 10.1021/acsphyschemau.6c00069

**Published:** 2026-08-03

**Authors:** Juan Soto

**Affiliations:** † Department of Physical Chemistry, Faculty of Science, University of Málaga, Andalucía Tech. E-29071, Málaga, Spain; ‡ University Institute of Materials and Nanotechnology (IMANA). University of Málaga, Campus de Teatinos E-29071, Málaga, Spain

**Keywords:** photochemistry, aryl trifluoromethyl diazirines and diazoalkanes, MS-CASPT2, CASSCF, DFT

## Abstract

The photochemical formation of carbenes from 3-phenyl-3-(trifluoromethyl)-3H-diazirine (**1**) and its diazo isomer, phenyl-trifluoromethyl-diazomethane (**2**), was investigated using high-level multiconfigurational wave function methods (MS-CASPT2) and density functional theory (DFT). Vertical excitation energies and the vibronic absorption spectrum of **1** were computed, confirming that the experimental signals reported in the literature originate from a single conformer. Our results demonstrate that *S*_1_ excitation in both isomers leads to ultrafast nonradiative deactivation through a shared *S*_1_/*S*_0_ conical intersection (CI1). In the condensed phase, this relaxation triggers a branching mechanism: the system can either revert to the precursor, undergo diazirine–diazo isomerization, or fragment into *N*_2_ and a closed-shell singlet carbene. Notably, the topology of the ground-state potential energy surface suggests that CI1 is a primary connection between the two isomers, relegating a conventional transition-state-mediated pathway to a less likely alternative for isomerization. Furthermore, a comparative assessment shows that both MS-CASPT2 and DFT provide consistent descriptions of the critical *S*_1_/*S*_0_ and *S*_0_/*T*_0_ crossing geometries. These findings provide a comprehensive mechanistic framework for the photolysis of aryl-trifluoromethyl diazirines.

## Graphical abstract

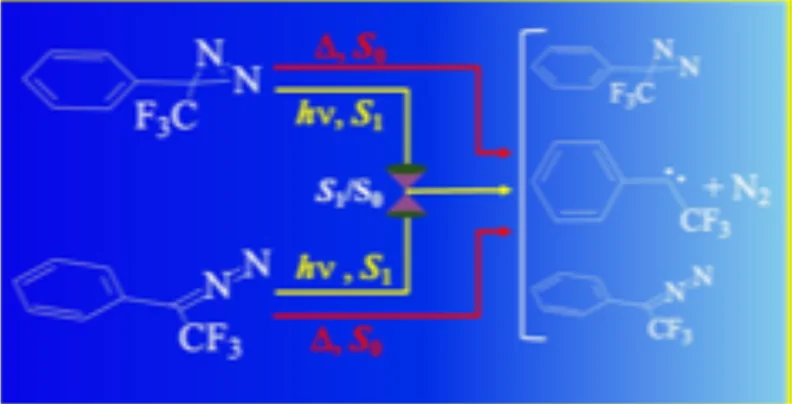

## 1 Introduction

Diazirines and disubstituted diazoalkanes are primarily utilized as carbene sources; while the former are frequently employed as photolabeling reagents, the latter find extensive use in synthetic organic chemistry. Within these families, aryl trifluoromethyl diazirines and diazoalkanes are particularly significant due to their ease of preparation and synthetic versatility. Consequently, their application in complex synthetic pathways is expanding rapidly.^1^ The pioneering synthesis of an aryl trifluoromethyl diazirine (3-phenyl-3-(trifluoromethyl)-3H-diazirine) was reported by Brunner et al., who utilized it as a carbene-generating group for photolabeling.^2^

From our perspective, the defining characteristic of aryl trifluoromethyl diazirines and diazoalkanes is their ability to generate free fluorinated carbenes via photochemical or thermal pathways (Scheme 1). A notable feature of these fluorinated carbene intermediates is that they are not deactivated by intramolecular proton migration, which would otherwise yield alkene derivatives.^3–5^ Photochemical decomposition remains the most common method for carbene generation from diazirines or diazoalkanes, a process fundamentally governed by an *S*_1_/*S*_0_ conical intersection. Due to the inherent nature of these excited-state processes, wave function-based quantum chemical methods are the most suitable for their description. Although the photochemistry of the low-lying excited states of small diazirines and diazo derivatives has been studied using CASSCF and MS-CASPT2,^6–8^ such methods have not yet been applied to 3-phenyl-3-(trifluoromethyl)-3H-diazirine (**1**) and its diazo isomer (phenyl-trifluoromethyl-diazomethane [**2**]). Accordingly, a computational study of these compounds was conducted using CASSCF and MS-CASPT2, complemented by a comparison with DFT-derived data.

**Scheme 1:**
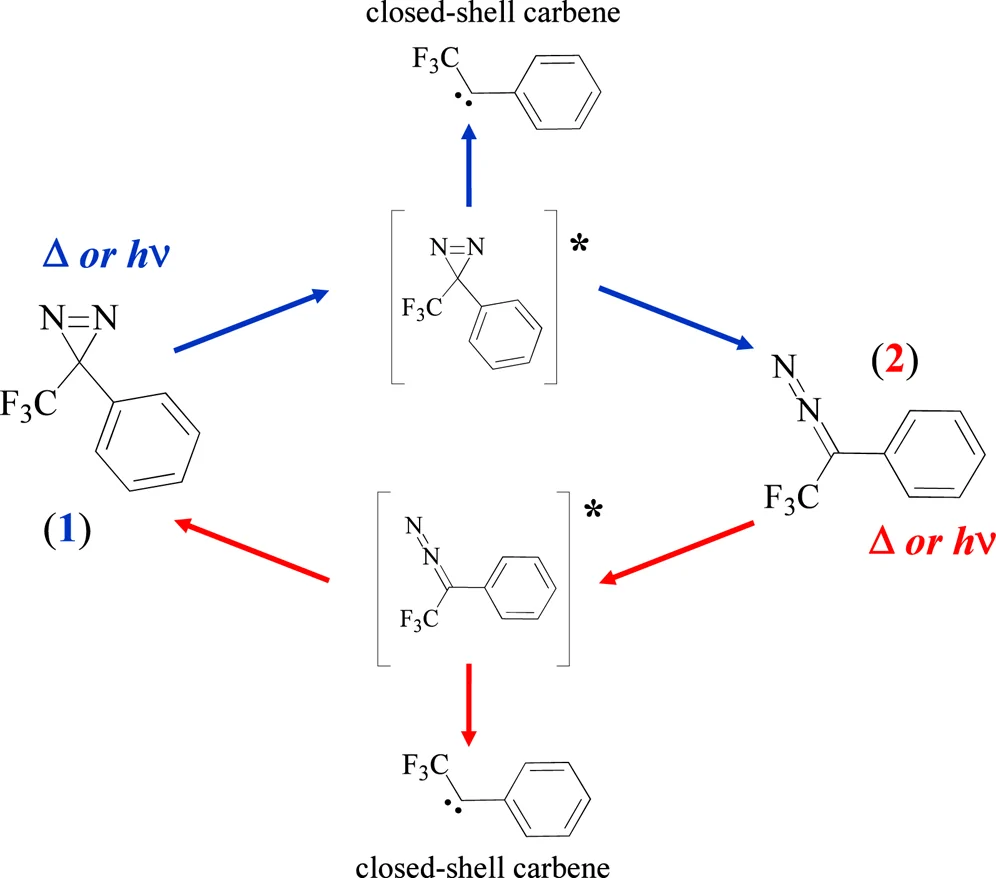
General Diagrammatic Representation of the Photochemical Decomposition Mechanism of **1** and **2**

The manuscript is organized as follows: Section 2 details the computational methodology, while Section 3 examines the electronic structure and excitation properties of **1** and its diazo isomer **2**, including a benchmark of the **1** absorption spectrum against experimental data. Section 4 explores the photochemical pathways, followed by an analysis of the energetic and structural features of the resulting products of the reaction (carbenes) and their interconversion in Section 5. Section 6 provides a rigorous comparative study of the critical points, evaluating the performance of density functional theory (DFT) against high-level multiconfigurational wave function methods (MS-CASPT2). Finally, concluding remarks are presented in Section 7.

## 2 Computational Details

Wave function-based calculations were performed using the Molcas 8.6 and OpenMolcas 24.06 packages,^9–12^ while density functional theory (DFT) calculations were conducted with ORCA 6.1.1.^13^ Geometries of the stable species were optimized at both the CASPT2 ^14,15^ and CAM-B3LYP^16^ levels of theory. For the CASPT2 calculations, the ANO-RCC basis set^17,18^ was employed with the C,F,N­[4*s*,3*p*,2*d*,1*f*]/H­[3*s*,2*p*,1*d*] contraction scheme, whereas the CAM-B3LYP optimizations utilized the Karlsruhe basis sets.^19,20^ Conical intersections were optimized using two approaches: CASSCF, by calculating the branching space vectors,^21^ and DFT, following the method developed by Maeda et al.^22^ In the DFT framework, the spin-flip formalism^23–25^ was employed to reasonably describe the *S*_1_/*S*_0_ intersections. The vibronic absorption spectrum^26,27^ of **1** was computed at the DFT level using the CAM-B3LYP hybrid functional, interfaced via an external library.^28^

To account for dynamic correlation, we applied MS-CASPT2 ^29^ and XDW-CASPT2 ^30,31^ methods to the CASSCF reference energies. In both cases, an IPEA shift of 0.25 au was applied. For the MS-CASPT2 calculations, imaginary shifts of 0.1 au were used to avoid intruder state singularities. For the XDW-CASPT2 calculations, an exponential factor ζ = 10^–8^ was utilized, with correction weights derived from the ratio of the square of the total energy of the state to the Hamiltonian coupling between states, and nor imaginary neither real imaginary shift was applied. Unless otherwise specified, all reported energies refer to the total or relative electronic energies, excluding zero-point vibrational (ZPE) or further thermodynamic corrections.

A critical aspect of this work is the state-averaging procedure. To address this, we conducted a systematic benchmark of different averaging schemes involving up to five states. As shown in Tables S1–S4 of the Supporting Information, an averaging of four states provides a robust and physically sound description of the electronic character of all states involved in the studied reactions. This choice is further validated by the smooth continuity of the potential energy curves along the reaction coordinates, confirming the stability of the wave functions obtained with this protocol.

One-dimensional potential energy surfaces (1D-PES) were constructed via linear interpolation in internal coordinates (LIIC).^32–34^ The multiconfigurational treatment for both the diazirine (**1**) and diazoalkane (**2**) forms and all the intermediates employed an active space of 14 electrons in 14 orbitals, denoted as CASSCF­(14e,14o). The specific orbitals of **1** and **2** and conical intersection (CI1) included in these active spaces are illustrated in Figures S1–S3. Geometric analysis and orbital visualizations were performed using Molden,^35^ MacMolPlt,^36^ and Gabedit.^37^

Regarding the active space of the diazo isomer, it is worth noting that it was impossible to simultaneously retain the σ*­(NCN) orbital of this species. Furthermore, various attempts to adjust the active space size to maintain its continuity along reaction coordinates were unsuccessful. To clarify this behavior, Figure S2b has been included in the Supporting Information, which displays the orbitals of the diazo species obtained from a single-root calculation for the ground state of **2** (where the σ*­(NCN) orbital is present). The corresponding orbital occupation numbers, also provided in this figure, demonstrate that the occupation of the σ*­(NCN) orbital in the single-state calculation is nearly zero (less than 0.01). This very low occupation explains why it becomes unfeasible to retain the σ*­(NCN) orbital in the active space when extending the state-averaged calculation to multiple roots. While maintaining absolute active-space continuity remains a challenge at the CASSCF level, the subsequent MS-CASPT2 calculations include dynamic correlation, which helps to smooth out these energetic descriptions and provides a more qualitatively consistent potential energy profile.

To ensure clarity and avoid ambiguity when discussing transitions between singlet and triplet manifolds, electronic states are labeled according to a reactivity-based convention. Specifically, both singlet and triplet states are indexed starting from zero (e.g., *S*_0_, *S*_1_,···and *T*_0_, *T*_1_,···).

## 3 Electronic Transitions and Absorption Spectrum

The absorption spectrum of the diazirine compound^2^ (**1**) displays a broad, intense band spanning the 400–300 nm range. This band exhibits multiple peaks, including a signature resonance at ∼365 nm characteristic of the diazirine chromophore. Given that **1** possesses two nearly free rotors—the trifluoromethyl and phenyl groups—it is crucial to determine whether these spectral features stem from a partially resolved vibrational structure or from a distribution of distinct conformers. To address this, we first evaluated the vertical transitions of both **1** and its diazo isomer (**2**) using MS-CASPT2 and CAM-B3LYP (Table 1). Subsequently, we computed the CAM-B3LYP absorption spectrum of **1** associated with the allowed *S*_0_ → *S*_1_ transition.

**Table 1:** Calculated Vertical Excitation Energies of the Two Lowest Singlet States of Two Conformers of 3-Trifluoromethyl-3-phenyldiazirine [**1**] and Phenyltri-fluoromethyldiazomethane [**2**]­^a^

isomer	state	Δ*E* ^b^	*f*_OSC_ ^c^	conf^d^	w^e^
**1** (MS-CASPT2)^f^	S_1_	3.68 (337)^f^	5.93 × 10^–03^	[π_3_(bz)]^1^[π*(NN)]^1^	0.74
	S_2_	4.86 (255)	1.50 × 10^–03^	[π_2_(bz)]^1^[π_3_*(bz)]^1^	0.32
				[π_2_(bz)]^1^[π*(NN)]^1^	0.16
	S_3_	5.64 (220)	1.50 × 10^–02^	[π_2_(bz)]^1^[π*(NN)]^1^	0.57
**1** (CAM-B3LYP)	S_1_	3.66 (339)	7.25 × 10^–03^	[π_3_(bz)]^1^[π*(NN)]^1^	0.74
				[σ_2_(NCN)]^1^[π*(NN)]^1^	0.22
	S_2_	5.30 (233)	2.05 × 10^–03^	[π_2_(bz)]^1^[π_3_*(bz)]^1^	0.15
				[π_3_(bz)]^1^[π_2_*(bz)]^1^	0.18
				[π_2_(bz)]^1^[π*(NN)]^1^	0.47
	S_3_	5.65 (219)	7.25 × 10^–03^	[π_2_(bz)]^1^[π*(NN)]^1^	0.44
				[π_2_(bz)]^1^[π_3_*(bz)]^1^	0.27
				[π_3_(bz)]^1^[π_2_*(bz)]^1^	0.20
**2** (MS-CASPT2)^f^	S_1_	2.82 (440)	-	[*n*_π_(C–N)]^1^ [π_2_*(NN)]^1^	0.72
	S_2_	4.64 (267)	1.35 × 10^–03^	[*n*_π_(C–N)]^1^ [π_2_*(bz)]^1^	0.26
				[π_2_(bz)]^1^[π_3_*(bz)]^1^	0.33
	S_3_	5.72 (217)	4.34 × 10^–05^	[π_3_(bz)]^1^[π_2_*(NN)]^1^	0.36
				[*n*_π_(C–N)]^0^[π_3_*(bz)]^1^[π_2_*(bz)]^1^	0.26
**2** (CAM-B3LYP)	S_1_	2.78 (446)	9.81 × 10^–06^	[*n*_π_(C–N)]^1^ [π_2_*(NN)]^1^	0.88
	S_2_	5.03 (247)	4.24 × 10^–03^	[π_2_(bz)]^1^[π_3_*(bz)]^1^	0.22
				[*n*_π_(C–N)]^1^ [π_3_*(bz)]^1^	0.17
				[*n*_π_(C–N)]^1^[π_2_*(bz)]^1^	0.53
	S_3_	5.29 (235)	4.87 × 10^–01^	[*n*_π_(C–N)]^1^ [π_3_*(bz)]^1^	0.73

a No symmetry was applied.

b Δ*E* in electron-volts.

c Oscillator strength (position representation).

d Only individual configurations with weights ≥0.15 are reported.

e Weight of the configuration ^f^Reference wave function: SA4-CASSCF/ANO-RCC.

f In parentheses, the wavelength is given in nm.

Table 1 summarizes the vertical excitation energies for the **1** and **2** isomers, calculated at the ground-state equilibrium geometry using both the MS-CASPT2 and CAM-B3LYP methods. The two levels of theory yielded consistent results for the first excited state (*S*_1_) of each isomer, particularly regarding excitation energies and the character and weights of the electronic configurations. In contrast, the agreement between the methods diminishes for the *S*_2_ and *S*_3_ states. This divergence reflects the greater complexity of describing higher-lying electronic states. Under these circumstances, it is difficult to assert that MS-CASPT2 results are inherently more reliable than those obtained via DFT, particularly given the presence of nine occupied nonbonding orbitals (the lone pairs of the fluorine atoms) that are not included in the active space, whose dynamical correlation remains unaccounted for in the multiconfigurational treatment.

Figure 1 displays the theoretical (A) and experimental^2^ (B) absorption spectra associated with the *S*_0_ → *S*_1_ transition of **1**. The calculated spectra have been obtained without the incorporation of solvent effects and use two distinct homogeneous bandwidths Γ = 100 cm^–1^ (red) and 500 cm^–1^ (black). The agreement between the theoretical and experimental spectra confirms that the observed signals originate from the **1** species. The theoretical spectra incorporate both Franck–Condon (FC) and Herzberg–Teller (HT) contributions. Since diazirine exhibits a local minimum in its first singlet excited state, the FC factors were determined using dimensionless displacement parameters derived from the geometry optimization differences between the *S*_0_ and *S*_1_ states. The assignment of the individual vibronic peaks follows the notation of Santoro and co-workers.^38^ Based on the labels in Figure 1, the transitions are assigned as follows: 1 [*S*_1_(**0**); (0–0 transition; (367 nm)], 2 [*S*_1_(43^1^); (346 nm)], 3 [*S*_1_(42^1^,43^1^); (327 nm)], 4 [*S*_1_(42^1^,43^2^); (310 nm)], and 5 [*S*_1_(35^1^,42^1^,43^2^); (298 nm)]. In this notation, the numbers between parentheses indicate the active normal mode labeled in Figure 1c, while the superscripts represent the corresponding vibrational quantum numbers. The calculated spectrum of diazo isomer is represented in Figure S4.

**Figure 1:**
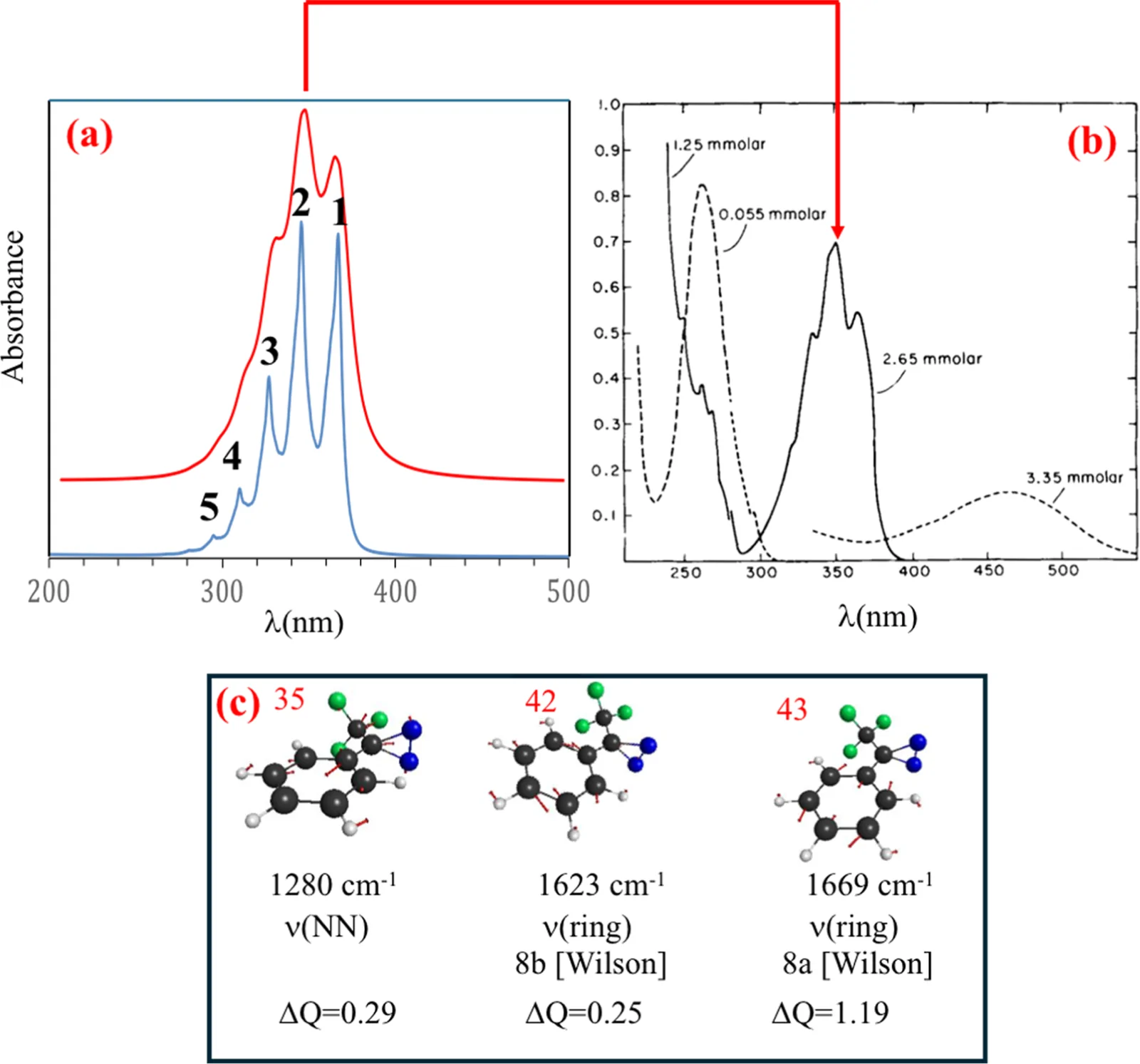
(a) Calculated absorption spectra of the *S*_1_ state of **1** with Γ = 500 cm^–1^ (red) and Γ = 100 cm^–1^ (blue). (b) Observed spectra of **1** (solid lines) and **2** (dashed lines). Reprinted under a Creative Commons CC BY 4.0 License from ref 2. Copyright 1980 American Society for Biochemistry and Molecular Biology. Published by Elsevier Inc. (c) Active modes in bands (2–5); see the text. Adiabatic difference energy: 27816 cm^–1^ (360 nm).

## 4 Photochemistry of 3-Trifluoromethyl-3-phenyldiazirine (1) and Phenyl-trifluoromethyldiazomethane (2)

For clarity, Figure 2 presents the minimum-energy crossing geometries found within the **1**–**2** system prior to their detailed discussion.

**Figure 2:**
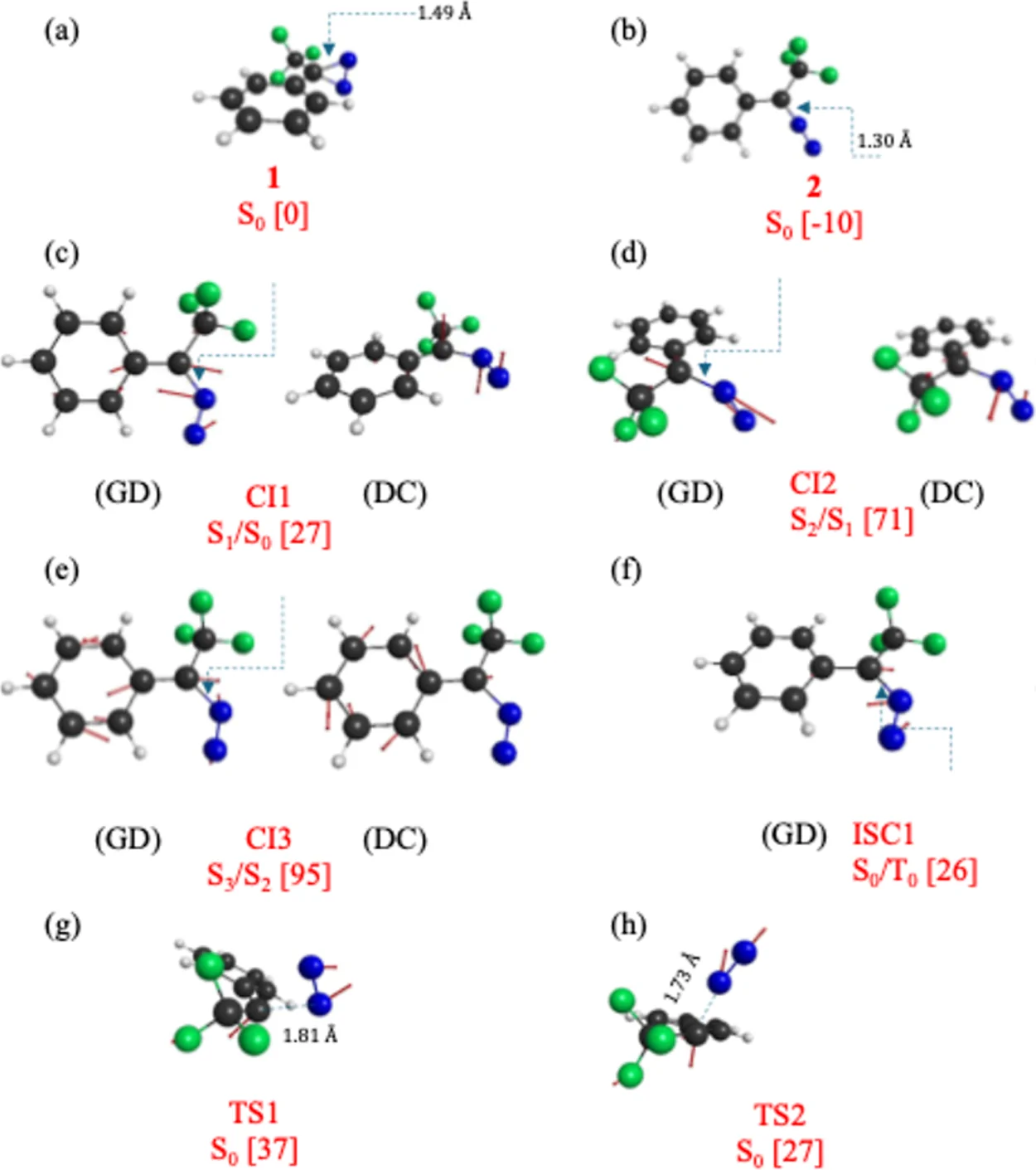
CASSCF geometries of the conical intersections and intersystem crossings found in this work. GD: gradient difference vector; DC: nonadiabatic coupling vector. In square brackets: MS-CASPT2 relative energies in kcal/mol.

The photochemical pathways of **1** and **2** in their first excited states are illustrated in Scheme 1, with their electronic properties summarized in Table 1. While the *S*_0_ → *S*_1_ transition exhibits a significant oscillator strength (*f*), it is negligible (*f* < 10^–6^) for the diazo compound. Consequently, the *S*_1_ state of the latter is accessible only via the Herzberg–Teller mechanism, requiring high-intensity radiation. In this section, we analyze the potential energy profiles connecting **1** and **2** (Figure 3). In accordance with previous semiclassical dynamical studies^6^ and experiments^39^ on 3H-diazizirine, our study permits to assert that the Franck–Condon excitation of the diazirine *S*_1_ state occurs at ∼337 nm, leading to a shallow minimum that is easily surmounted upon irradiation. Subsequently, the system undergoes ultrafast decay on a femtosecond time scale through a peaked *S*_1_/*S*_0_ conical intersection (**CI1**). Relaxation through this conical intersection leads directly to the *S*_0_ minima of either **1** or **2**. A similar line of reasoning applies to the diazoalkane. This branching decay channel has been previously predicted by theoretical studies.^6,7^ In the gas phase, once a specific minimum is populated, the system undergoes dissociation along the ground-state potential energy surface to yield the carbene derivative and molecular nitrogen on a picosecond time scale. This mechanism was recently corroborated by Datta and Davis for the simplest diazirine,^39^ where no evidence for the triplet channel was found on the picosecond time scale. Thus, our results suggest that the primary dissociation pathways for **1** and **2** on the ground-state potential energy surface are mediated by **TS1** (37 kcal/mol) and **TS2** (27 kcal/mol), respectively, both of which yield singlet closed-shell carbene (intrinsic reaction coordinates starting at each transition state are included in Figure S5). Nevertheless, it should be noted that Blanch and Wentrup^40^ observed the facile formation of triplet phenyl-trifluoromethylcarbene upon photolysis of both diazirine and diazo precursors, a finding consistent with the nanosecond lifetime of the singlet carbene species.^41^

**Figure 3:**
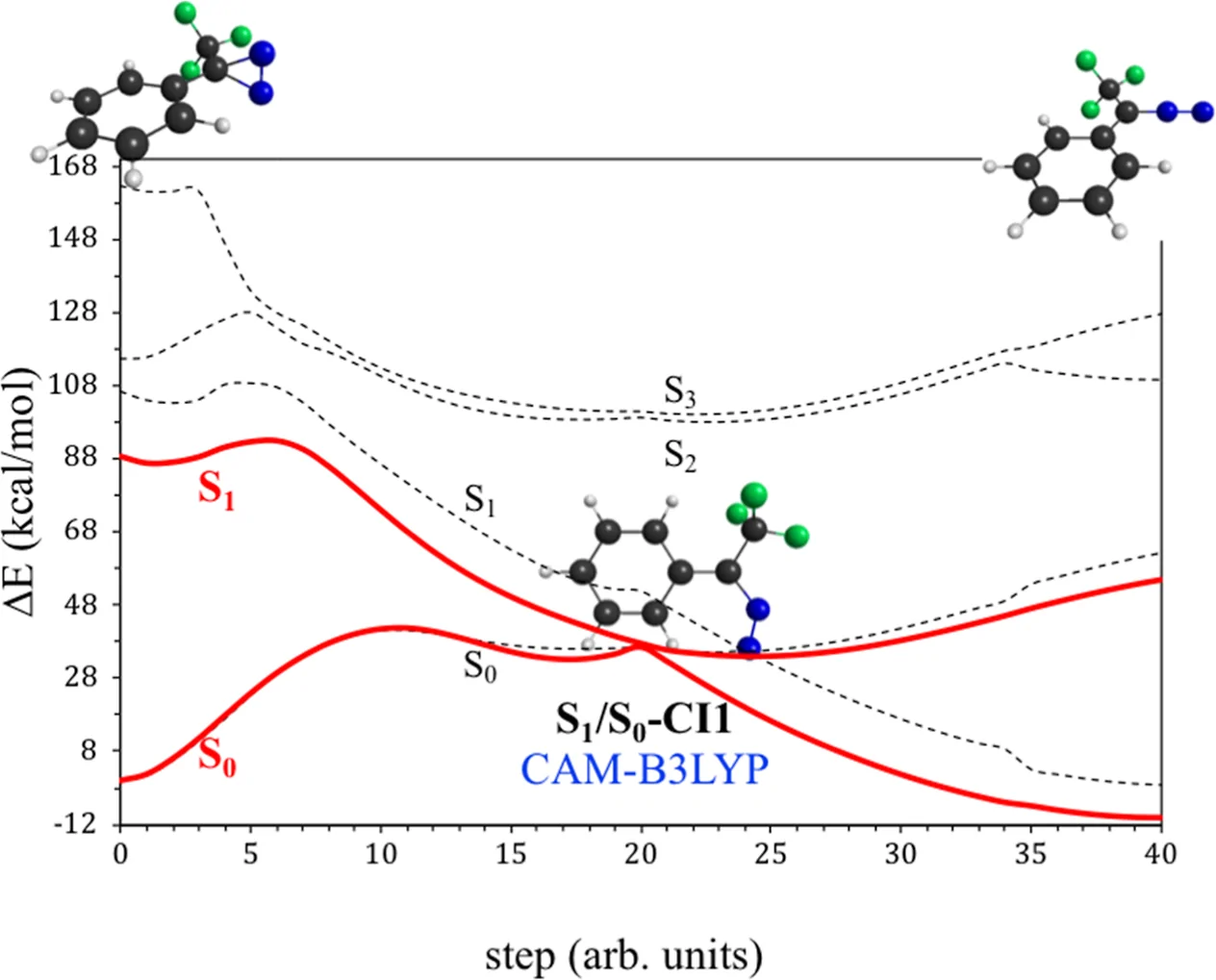
Energy profiles of the lowest singlet states calculated at the CASSCF (dashed lines) and MS-CASPT2 (solid lines) levels of theory. The linear interpolated internal coordinate (LIIC) path connects the diazirine, ***S***_1_ **/*S***_0_ **–CI** and diazoalkane geometries. The initial and final points correspond to the CASPT2 *S*_0_ geometries of **1** and **2**, respectively; the middle point corresponds to the CAM-B3LYP ***S***_1_ **/*S***_0_ **–CI** optimized geometry.

An alternative dissociation pathway into carbene and N_2_ involves the formation of the triplet state of **1** or **2** to yield the most energetically stable species of phenyl-trifluoromethyl carbene, the triplet carbene. Given that this process requires a singlet–triplet intersystem crossing (ISC), we identified the minimum energy crossing point (**ISC1** (Figure 2), 22 kcal/mol, SOC = 25.5 cm^–1^) between the *S*_0_ and *T*_0,_ the potential energy surfaces. Notably, this crossing point is shared by both the diazirine and diazoalkane species, in analogy with the **CI1** conical intersection. Furthermore, the geometry of this crossing point (**ISC1**) is remarkably close to the **CI1** conical intersection, with calculated distances between these two points of only 0.11 (CASSCF) and 0.23 Å (CAM-B3LYP). This proximity has profound mechanistic and dynamical implications: the ISC mechanism is significantly hindered by the topological constraints imposed by the adjacency of **ISC1** to **CI1**. Specifically, while the peaked *S*_1_/*S*_0_ (CI1) conical intersection is attractive for the *S*_1_ → *S*_0_ transition, it becomes repulsive when approached from the opposite direction—the ground state—effectively diverting the trajectory away from the **CI1–ISC1** region (Figure S6). This behavior is supported by our previous molecular dynamics studies on 3H-diazirine,^6^ which demonstrated that upon passing through the *S*_1_/*S*_0_ conical intersection, the dissociating fragments (CH_2_ and N_2_) undergo a massive acceleration, with velocities increasing by orders of magnitude. This rapid acceleration reflects a strongly attractive potential energy surface in the *S*_1_ → *S*_0_ direction. Consequently, by microscopic reversibility and the topography of the ground-state surface, approaching this region from the opposite direction (*S*_0_ → *S*_1_) encounters a highly repulsive profile, which prevents the system from re-entering the CI1–ISC1 region.

In summary, the photochemistry of both isomers in the first excited state exhibits phase-dependent pathways after the *S*_1_ → *S*_0_ nonradiative decay. In the gas phase, both the diazirine and diazoalkane species undergo decomposition into singlet closed-shell carbene and molecular nitrogen, as the internal energy exceeds their respective dissociation barriers. In contrast, the dissipative environment of the solution phase facilitates vibrational relaxation at a rate competitive with dissociation to yield both isomers in their respective electronic ground states as it was observed by Brunner et al.^2^ This solvent-mediated energy transfer stabilizes the diazo intermediate or promotes internal conversion back to the diazirine ground state, as it was reported by Brunner et al.^2^

Furthermore, exploration of the *S*_3_ and *S*_2_ potential energy surfaces identified two additional electronic degeneracies: an *S*_2_/*S*_1_ conical intersection (**CI2**) and an *S*_3_/*S*_2_ conical intersection (**CI3**). The mechanistic pathways connecting these higher-order crossings with CI1 are illustrated in Figure 4a. In accordance with Figure 3, population of the *S*_3_ state in both isomers leads to a quasi-degenerate region where CI3 represents the lowest-energy point. Upon decaying to the *S*_2_ state, the system reaches CI2 after surmounting a small barrier. At this CI2 region, three competitive channels become energetically accessible: (1) sequential cascade to *S*_0_, i.e., excitation to the *S*_3_ state likely triggers a sequential decay toward the ground state via CI1, consistent with Kasha’s rule to give the products described previously. (2) Direct *S*_2_ dissociation to yield the carbene in its 2^1^Á excited state. This latter pathway cannot be entirely ruled out, as previously reported for related diazo compounds.^42,43^ (3) Direct dissociation into the *S*_1_ state given that the gradient difference vector within the branching space (*g-h* plane) of CI2 aligns with a dissociative coordinate, potentially yielding the open-shell carbene in its singlet state (1^1^A″).

**Figure 4:**
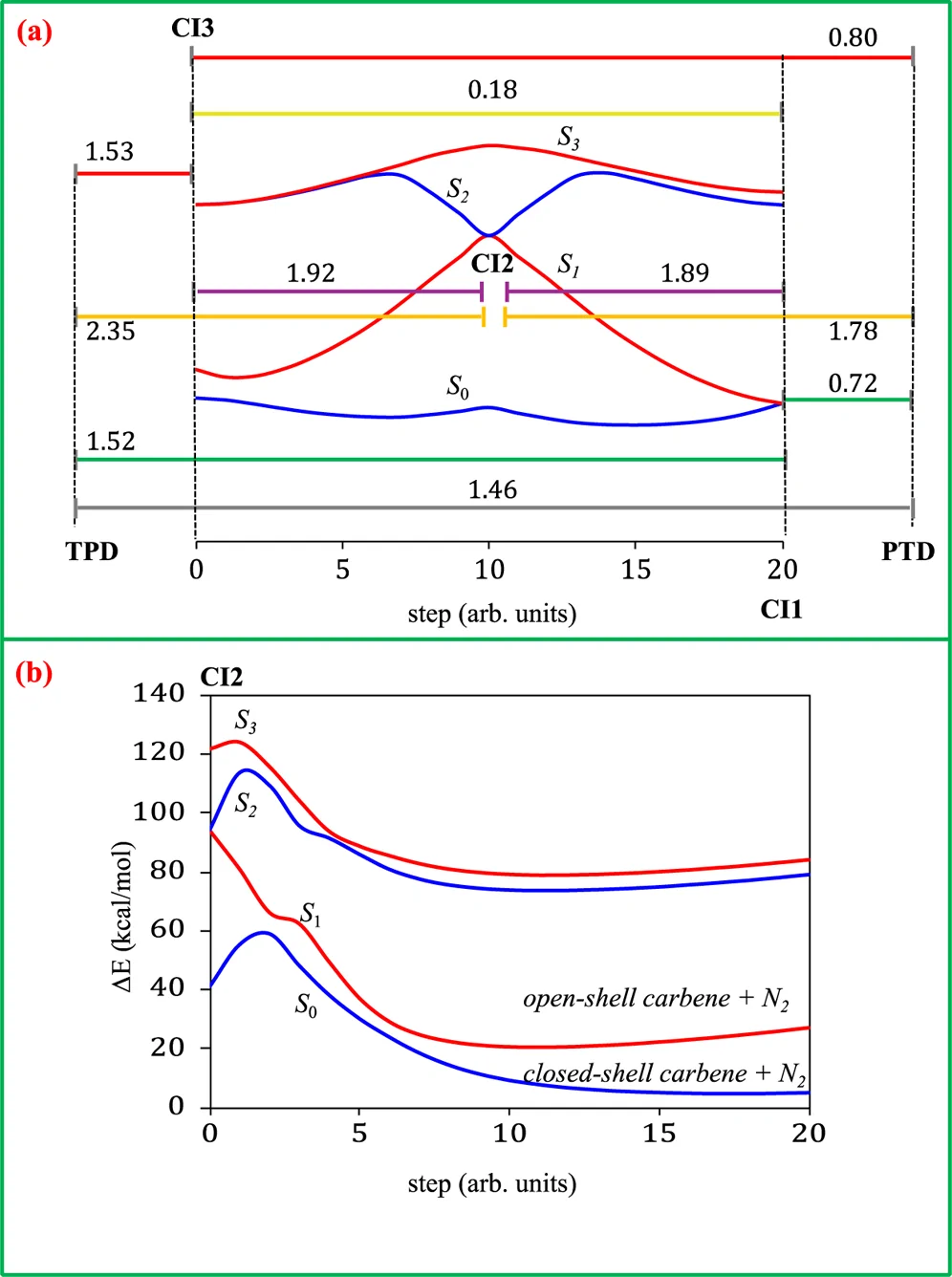
(a) CASSCF energy profiles along the linear interpolation in internal coordinate (LIIC) paths connecting the CI3, CI2, and CI1 conical intersections. Geometrical distances (dimension markings) are provided in Å. (b) CASSCF energy profiles for the dissociation into carbene and *N*_2_, initiated from the CI2 intersection. The curves illustrate the potential energy surfaces of the four lowest singlet states along the dissociative coordinate.

The dissociation pathways originating at CI2 are shown in Figure 4b. The potential energy profiles indicate that direct dissociation from the *S*_1_ state following an *S*_2_/*S*_1_ surface hop is a plausible route.

## 5 Electronic Structure and State Inter-Relations of Phenyl-trifluoromethyl Carbenes

The electronic manifold of phenyl-trifluoromethyl carbene follows the energy hierarchy *T*_0_ < *S*_0_ < *S*_1_. While the closed-shell singlet (*S*_0_) is the primary intermediate generated during the photolysis and thermolysis of aryl-trifluoromethyl diazirines and diazo compounds, the formation of the *S*_1_ state remains a distinct possibility. Furthermore, given that *S*_0_ lifetimes of carbenes,^41^ before decaying to the triplet manifold, are of the order of 0.1 ns, characterizing the energy profiles connecting these three states is essential. Figure 5a illustrates the one-dimensional potential energy surface (1D-PES) connecting the minima of both the *S*_1_ and *S*_0_ states calculated at the XDW-CASPT2 level. These singlet states are linked by a sloped conical intersection (**CI4**) situated near the *S*_1_ minimum, suggesting that the *S*_1_ carbene is inherently unstable and undergoes rapid internal conversion to *S*_0_. Conversely, Figure 5b displays the 1D-PES for the *S*_0_ and *T*_0_ states calculated at the same level of theory as Figure 5a. The calculated gap between both states is 3.4 kcal/mol in agreement with previous studies.^44–46^ The surface crossing occurs near the *S*_0_ minimum; optimization of this minimum energy crossing point (MECP) identifies **ISC2** at 0.4 kcal/mol above the *S*_0_ minimum, with a spin–orbit coupling (SOC) of 10.0 cm^–1^. Qualitatively, according to the Landau–Zener framework,^47–49^ the localization of ISC2 near the *S*_0_ minimum—where the nuclear velocity reaches its maximum—is expected to decrease the probability of intersystem crossing, thereby extending the lifetime of the *S*_0_ state.

**Figure 5:**
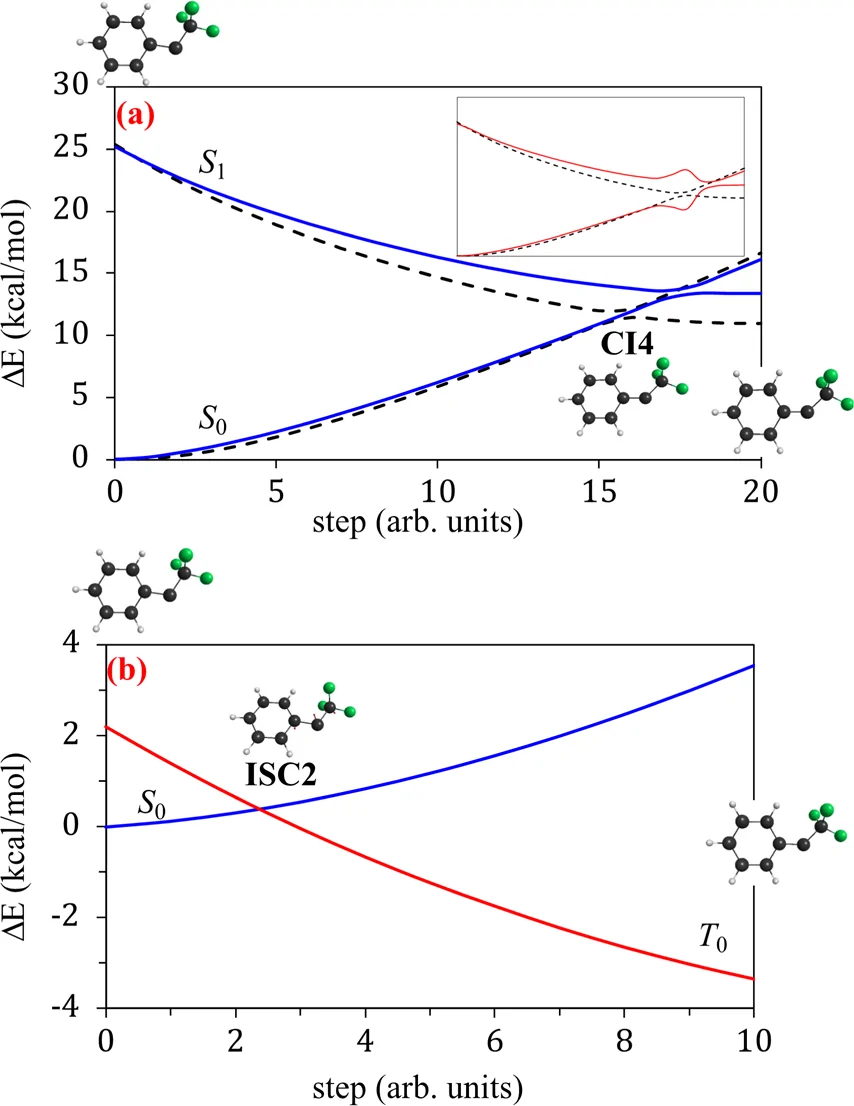
(a) XDW-CASPT2 energy profiles along the linear interpolation in internal coordinate (LIIC) paths connecting the *S*_0_ and *S*_1_ minimum energy geometries of the carbene. Inset: MS-CASPT2 profiles of the same LIIC. (b) XDW-CASPT2 energy profiles along the LIIC paths connecting the *S*_0_ and *T*_0_ minimum energy geometries of the carbene. Solid lines correspond to XDW-CASPT2 values and dashed lines to CASSCF values.

## 6 Comparative Assessment of WFT and DFT Methods for Characterizing Crossing Surfaces

In this section, we evaluate the performance of wave function theory (WFT) and density functional theory (DFT) in characterizing the geometries and relative energies of conical intersections and intersystem crossings for the systems under study. The results are summarized in Table 2.

**Table 2:**
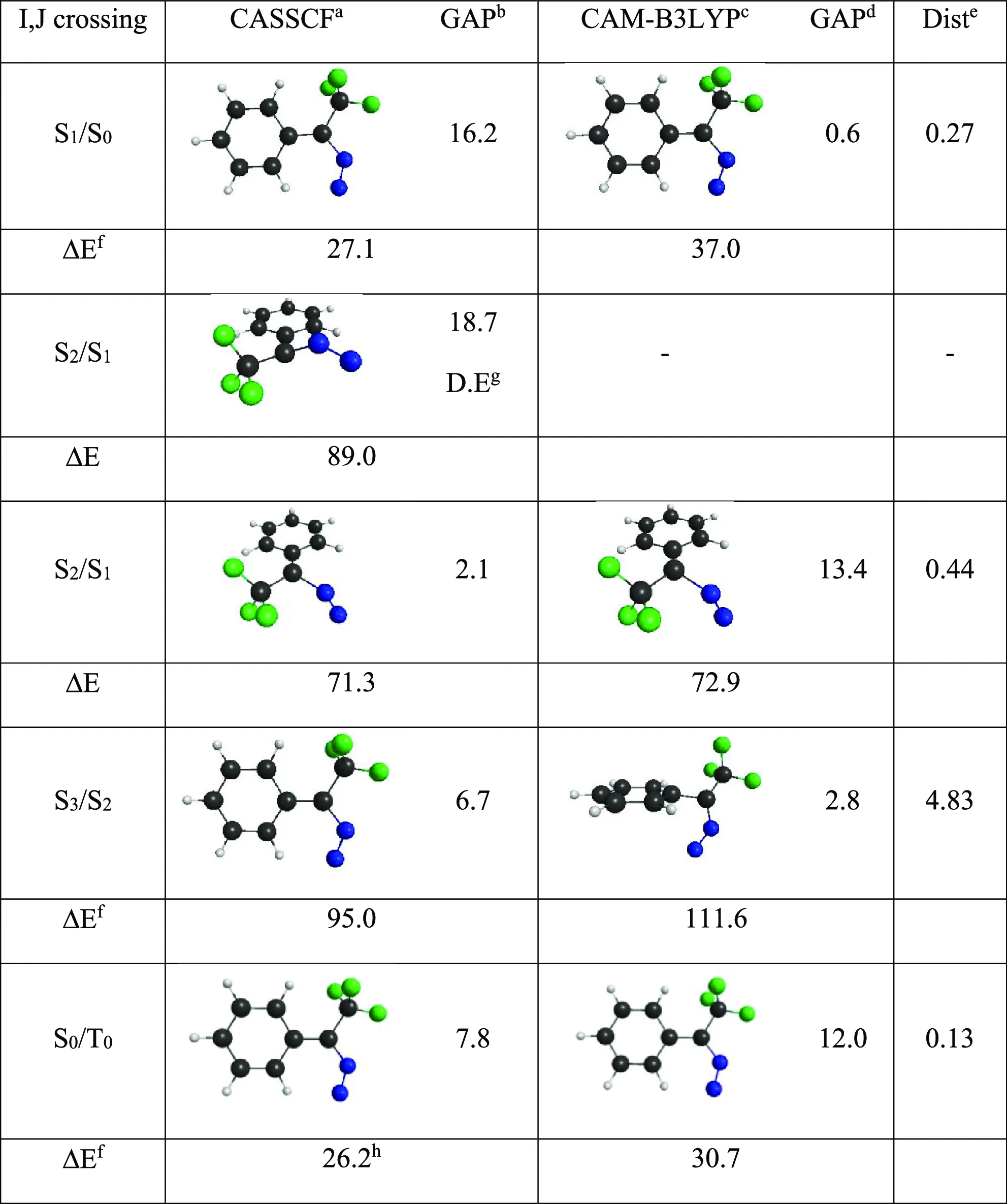
Energetics of the Minimum Energy Crossing Geometries Obtained with the CASSCF and CAM-B3LYP Methods^a^

a SA4-CASSCF­(14e,14o)/ANO-RCC.

b I,J MS-CASPT2 energy gap in kcal/mol at the CASSCF-optimized geometry of the crossing point.

c CAM-B3LYP/def2-TZVPP.

d I,J MS-CASPT2 energy gap in kcal/mol at the CAM-B3LYP-optimized geometry of the crossing point.

e Distance in Å with maximum alignment between CASSCF and CAM-B3LYP geometries in Cartesian coordinates.

f MS-CASPT2 relative energy in kcal/mol of the I,J mean value with respect to the diazirine minimum. ^g^ Double excitation.

h Calculated from the difference between the *S*_0_ electronic energies of CI1 and those of ISC1.

Overall, CAM-B3LYP with spin-flip formalism provides reasonably consistent results for selected low-lying crossing structures, particularly the *S*_1_/*S*_0_ intersections. However, it should be noted that this agreement is not uniform across all crossing regions, as evidenced by the larger discrepancies found for the higher energy *S*_3_/*S*_2_ crossing geometry and energy.

Since the crossing points were initially optimized at the CASSCF or DFT levels and subsequently the energies refined using MS-CASPT2, a critical metric is the energy gap between the two electronic states postcorrection. Regarding this gap, neither method demonstrates a systematic superiority; while CASSCF provides more accurate descriptions in certain cases, DFT performs better in others.

## 7 Conclusions

The photochemical decomposition pathways of **1** and **2** were systematically investigated using (MS)-CASPT2 and CAM-B3LYP levels of theory. Our findings reveal that *S*_1_ excitation in both species triggers nonradiative deactivation through a common conical intersection (CI1). In the condensed phase, this relaxation leads to a branching of pathways: the system may revert to the precursor, undergo diazirine–diazo isomerization, or fragment within the *S*_0_ state to yield *N*_2_ and the closed-shell singlet carbene via two isomer-dependent channels (Scheme 2). It should be noted, however, that this mechanism is expected to dominate on short time scales (subnanosecond), as longer-time dynamics may involve additional processes not covered in the present study.

**Scheme 2:**
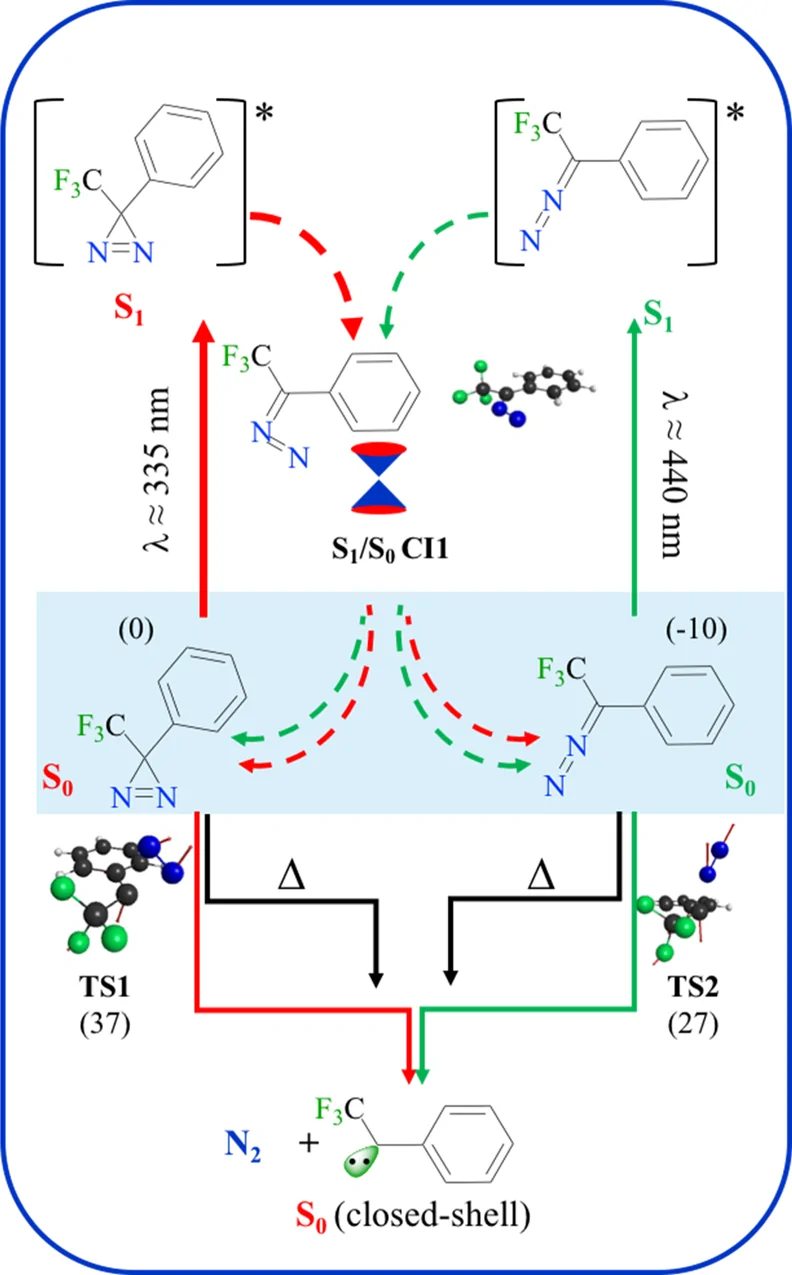
Mechanisms of the Photochemical Reactions of **1** and **2** after Population of Their First Excited States. Red and Green Arrows Correspond to Photochemical Processes, Whereas Black Arrows Correspond to Thermal Processes

At this point, it is worth noting that all our attempts to find a conventional reaction path passing through a transition state were unfruitful. Therefore, our current data suggest that CI1 represents a primary connection between the two isomers on the ground-state potential energy surface. In fact, this topography suggests that a conventional minimum energy path through a transition state for the diazirine–diazo isomerization is less likely, in a manner analogous to the nitro-nitrite isomerization of nitramide.^50^ Thus, we hypothesize that the process plausibly proceeds via a roaming mechanism rather than advancing through a traditional reaction valley.

Furthermore, our analysis demonstrates that the experimental absorption spectrum reported by Brunner et al. originates from a single conformer of **1**, providing a clearer interpretation of the available spectroscopic data. Finally, it is important to emphasize that both wave function-based and density functional theory methods yielded consistent and satisfactory crossing geometries, validating the robustness of the multilevel approach employed in this study.

## Supplementary Material



## Notes

Tables S1–S4, benchmarking of state-averaging schemes; Figure S1, SA4-CASSCF orbitals of **1**; Figures S2a and S2b, SA4-CASSCF and SS-CASSCF orbitals of **2**; Figure S3, SA4-CASSCF orbitals of CI1; Figure S4, CAM-B3LYP absorption spectrum of the S_1_ and S_2_ states of **2**; Figure S5, intrinsic reaction coordinates leading to carbene formation from **1** and **2**; Figure S6, schematic representation of CI1-ISC1 interaction; coordinates in Å and absolute electronic energies of all the featuring species of this work (PDF)
