# Supplementary material for: Mechanistic Insights into the Photochemical Formation of Carbenes from Phenyl-trifluoromethyl Diazirine and Its Diazo Isomer: An MS-CASPT2 Study
Source: ACS Phys Chem Au. 2026 Aug 3;6(5):1235–43. doi: 10.1021/acsphyschemau.6c00069 (PMC13614134; doi:10.1021/acsphyschemau.6c00069)
Supplement: Supplementary file 1 [file pg6c00069_si_001.pdf]

# *Mechanistic Insights into the Photochemical Formation of Carbenes from Phenyltrifluoromethyl Diazirine and its Diazo Isomer. An MS-CASPT2 Study*

*Juan Soto**[1,2]

[1]Department of Physical Chemistry, Faculty of Science, University of Málaga, Andalucía Tech., Málaga, Spain, E-29071.

[2]University Institute of Materials and Nanotechnology (IMANA). University of Málaga, Campus de Teatinos, Málaga, Spain, E-29071.

**Benchmarking of state-averaging schemes**

Tables S1-S4 present the vertical excitation energies of **1** calculated using different averaging schemes (involving up to five electronic states), obtained both with and without spatial symmetry.

**Table S1.** MS-CASPT2 vertical excitations (eV) of 3-trifluoromethyl-3-phenyldiazirine (**1**). Reference wave function: SA2-CASSCF(14e,14o)/ANO-RCC.

| State | ΔE NOSYM[a] | | ΔE SYMM[b] | |
|---|---|---|---|---|
| $S_0$ | 0.0 | - | | |
| $S_1$ | 4.84 | $(\pi_3)^1(\pi_3*)^1$; 33%<br>$(\pi_2)^1(\pi_2*)^1$; 33% | 4.84 | $(\pi_3)^1(\pi_3*)^1$; 33%<br>$(\pi_2)^1(\pi_2*)^1$; 33% |

[a]No symmetry applied. [b]A' symmetry.

**Table S2.** MS-CASPT2 vertical excitations (eV) of 3-trifluoromethyl-3-phenyldiazirine (**1**). Reference wave function: SA3-CASSCF(14e,14o)/ANO-RCC.

| State | ΔE NOSYM[a] | | ΔE SYMM[b] | |
|---|---|---|---|---|
| $S_0$ | 0.0 | - | | |
| $S_1$ | 3.54 | $(\pi_3)^1(\pi_{NN}*)^1$; 69% | 3.54 | $(\pi_3)^1(\pi_{NN}*)^1$; 69% |
| $S_2$ | 4.98 | $(\pi_2)^1(\pi_2*)^1$; 28%<br>$(\pi_3)^1(\pi_3*)^1$; 17% | 4.98 | $(\pi_2)^1(\pi_2*)^1$; 28%<br>$(\pi_3)^1(\pi_3*)^1$; 17% |

[a]No symmetry applied. [b]A' symmetry.

**Table S3.** MS-CASPT2 vertical excitations (eV) of 3-trifluoromethyl-3-phenyldiazirine (**1**). Reference wave function: SA4-CASSCF(14e,14o)/ANO-RCC.

| State | ΔE NOSYM[a] | NO SYMM | ΔE SYMM[b] | |
|---|---|---|---|---|
| $S_0$ | 0.0 | - | - | |
| $S_1$ | 3.68 | $(\pi_3)^1(\pi_{NN}*)^1$; 62% | 3.69 | $(\pi_3)^1(\pi_{NN}*)^1$; 66% |
| $S_2$ | 4.86 | $(\pi_2)^1(\pi_2*)^1$; 25%<br>$(\pi_3)^1(\pi_3*)^1$; 22% | 4.89 | $(\pi_2)^1(\pi_2*)^1$; 28%<br>$(\pi_3)^1(\pi_3*)^1$; 21% |
| $S_3$ | 5.64 | $(\pi_2)^1(\pi_{NN}*)^1$; 60% | 6.11 | $(\pi_2)^1(\pi_{NN}*)^1$; 38% |

[a]No symmetry applied. [b]A' symmetry.

**Table S4.** MS-CASPT2 vertical excitations (eV) of 3-trifluoromethyl-3-phenyldiazirine (**1**). Reference wave function: SA5-CASSCF(14e,14o)/ANO-RCC.

| State | ΔE NOSYM[a] | NO SYMM | ΔE SYMM[b] | |
|---|---|---|---|---|
| $S_0$ | 0.0 | - | - | |
| $S_1$ | 3.72 | $(\pi_3)^1(\pi_{NN}*)^1$; 64% | 3.72 | $(\pi_3)^1(\pi_{NN}*)^1$; 68% |
| $S_2$ | 4.83 | $(\pi_3)^1(\pi_3*)^1$; 21%<br>$(\pi 2)^1(\pi_2*)^1$; 28% | 4.83 | $(\pi_3)^1(\pi_3*)^1$; 17%<br>$(\pi 2)^1(\pi_2*)^1$; 25% |
| $S_3$ | 5.64 | $(\pi_2)^1(\pi_{NN}*)^1$; 59% | 5.64 | $(\pi_2)^1(\pi_{NN}*)^1$; 50% |
| $S_4$ | 6.07 | $(\sigma_{NCN})^1(\pi_{NN}*)^1$; 46% | 6.07 | $(\sigma_{NCN})^1(\pi_{NN}*)^1$; 45% |

[a]No symmetry applied. [b]A′ symmetry.

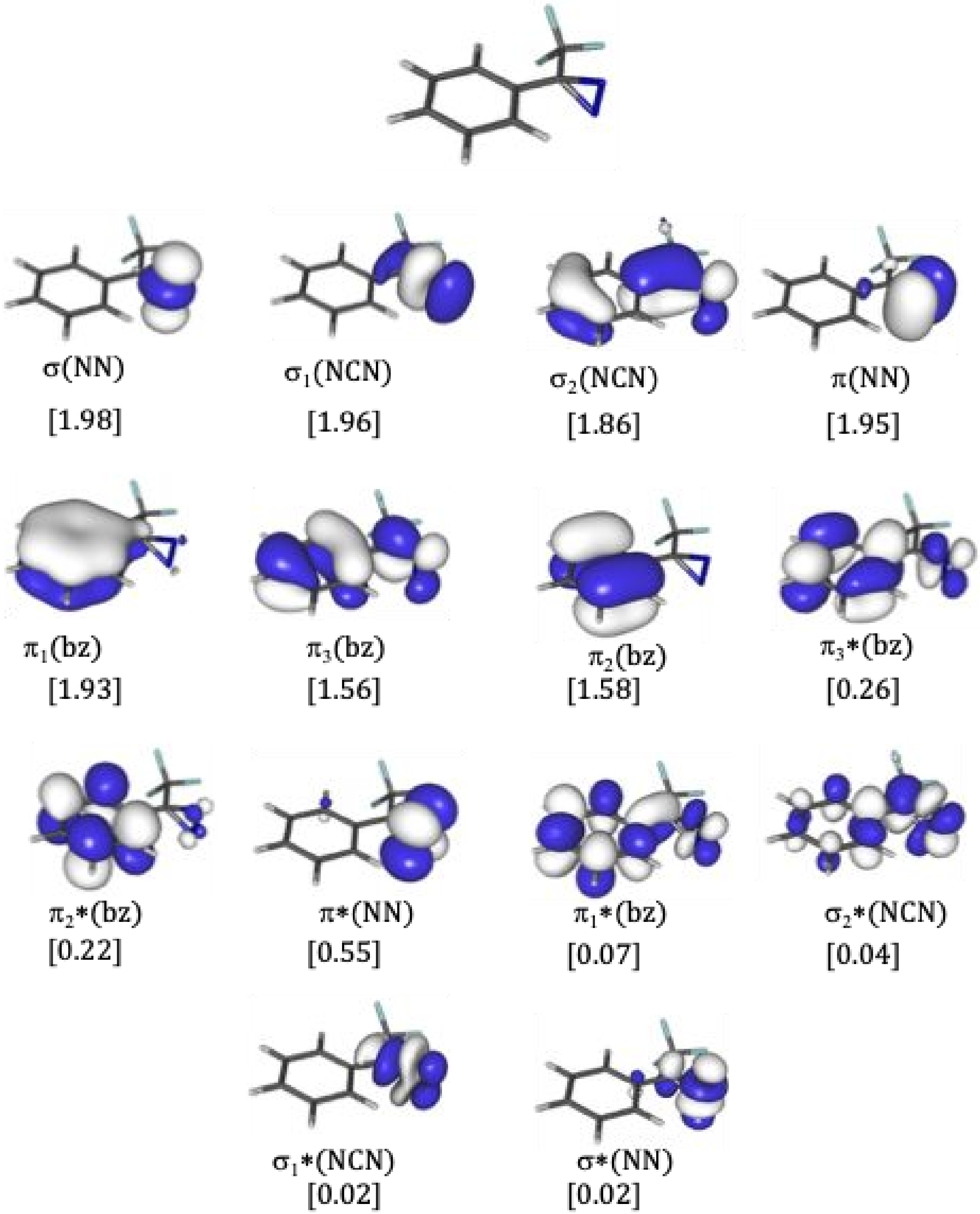


**Figure S1.** SA4-CASSCF orbitals included in the active space of 3-trifluoromethyl-3-phenyldiazirine (**1**). In square brackets, occupation numbers.

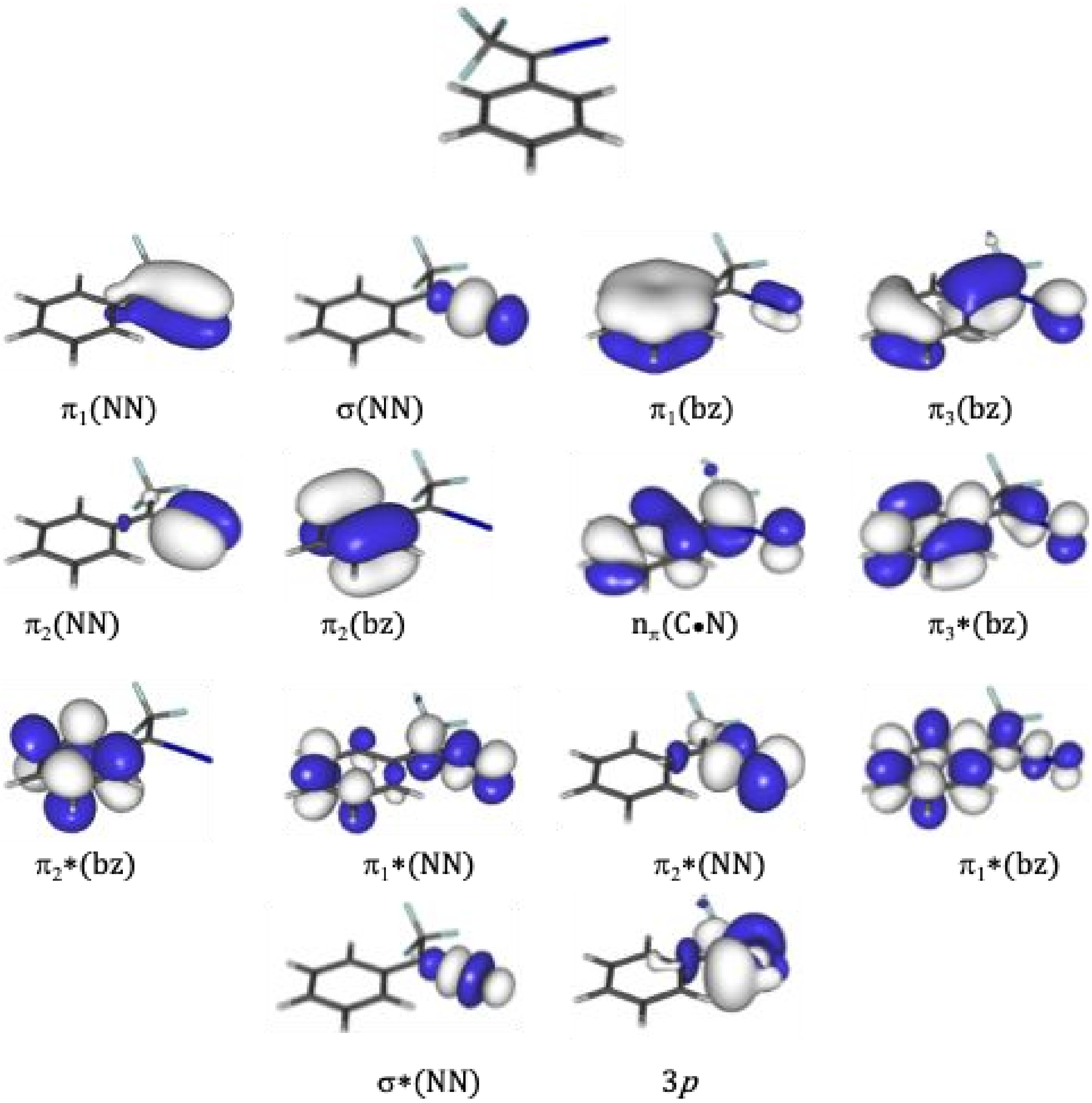


**Figure S2a.** SA4-CASSCF orbitals included in the active space of phenyl-trifluoromethyl-diazomethane (**2**). In square brackets, occupation numbers.

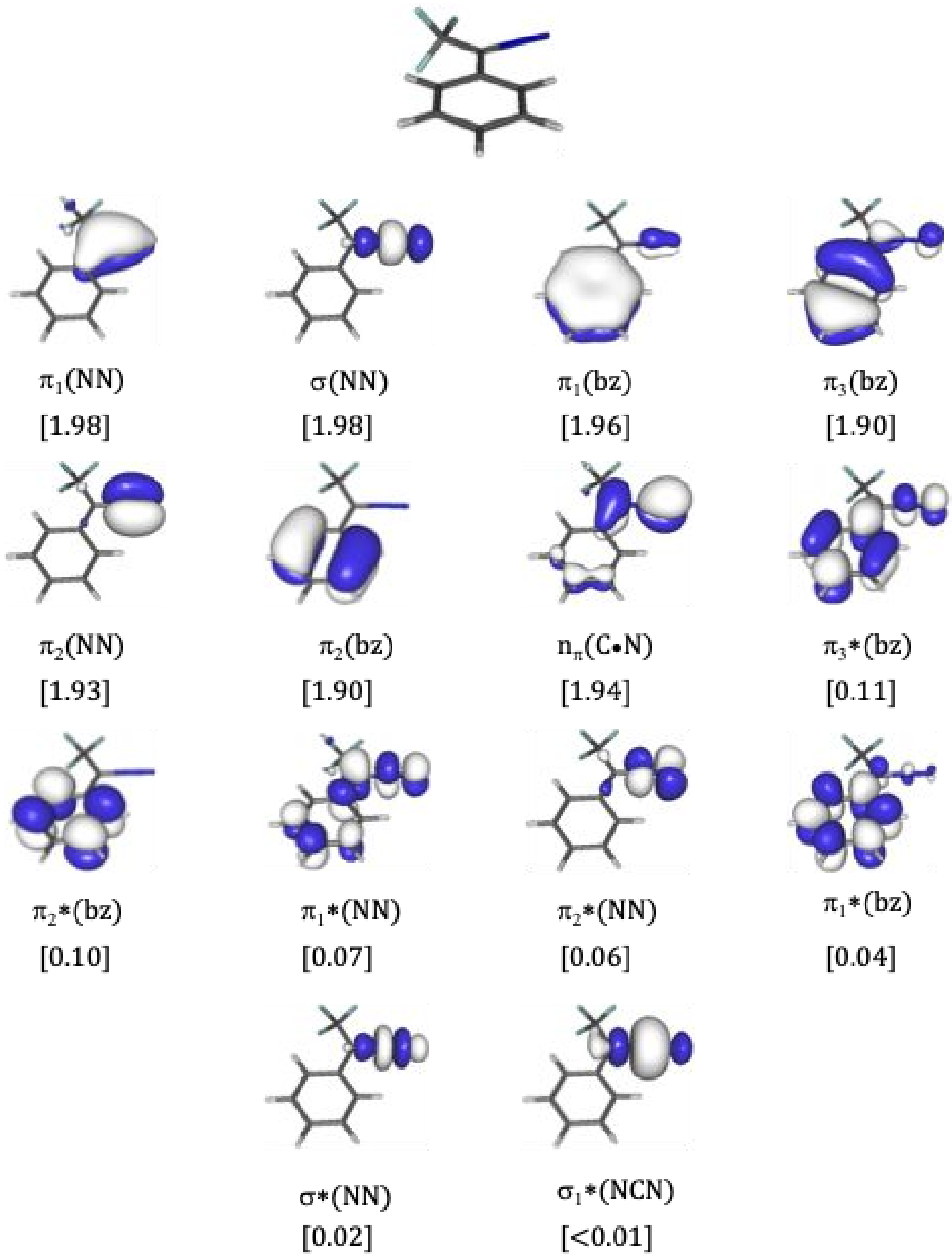


**Figure S2b.** SS-CASSCF orbitals included in the active space of phenyl-trifluoromethyl-diazomethane (**2**). In square brackets, occupation numbers.

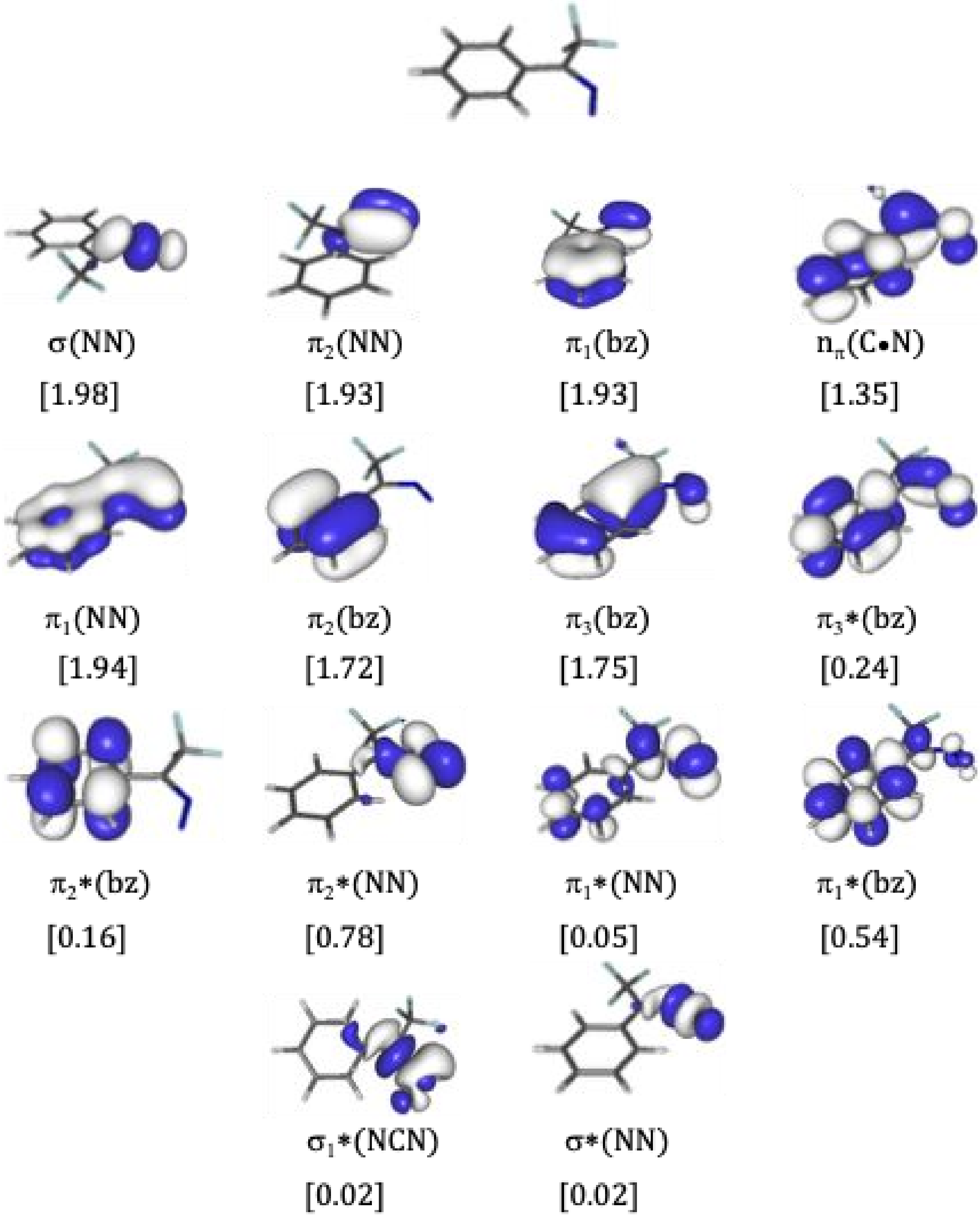


**Figure S3.** SA4-CASSCF orbitals included in the active space of **CI1**. In square brackets, occupation numbers.

(a)

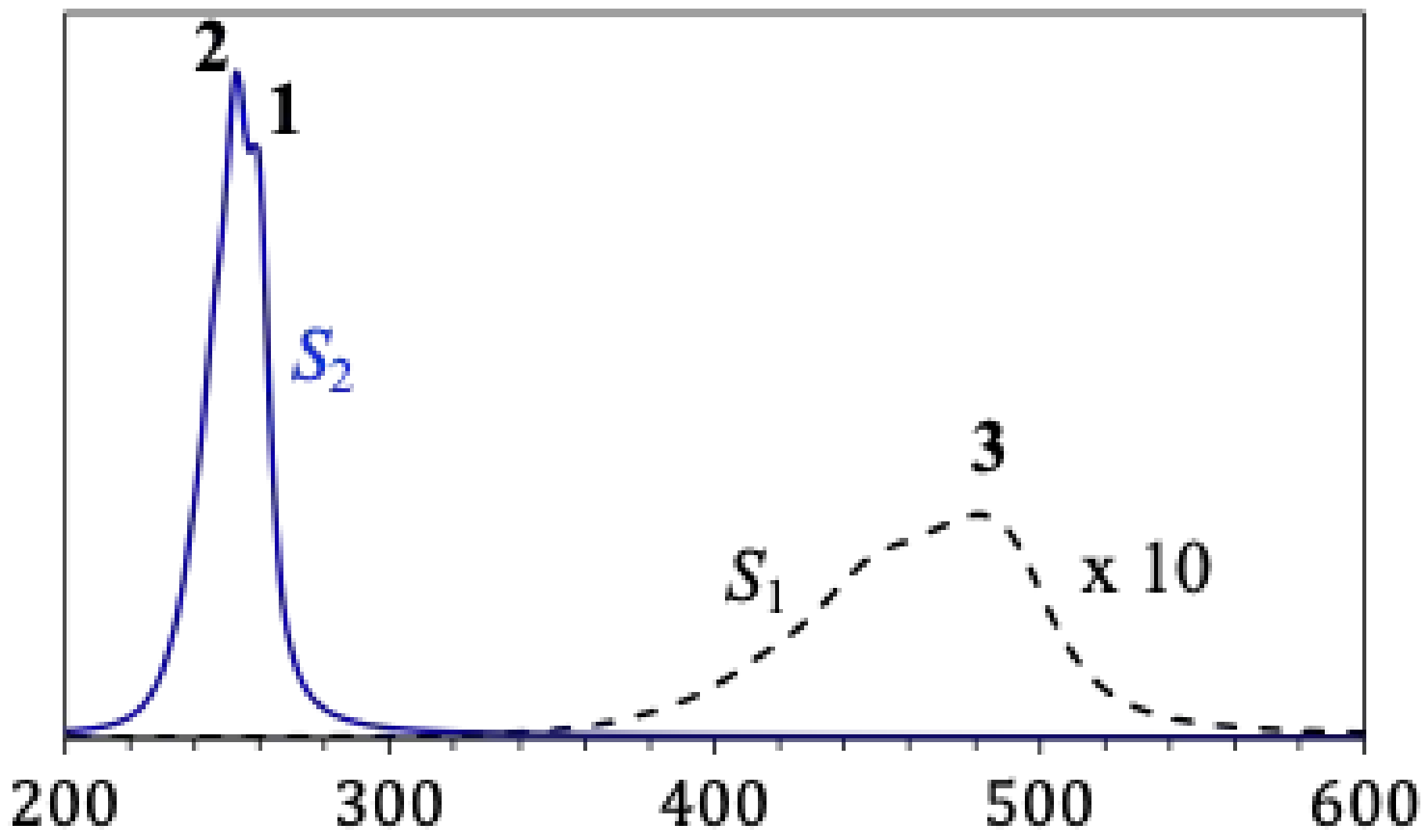


(b)

**1**: [$S_2$(**0**); (0-0 transition; (258 nm)]
**2**: [$S_2$(**26**); (252 nm)]
**3**: [$S_1$(**0**); (0-0 transition; (482 nm)]

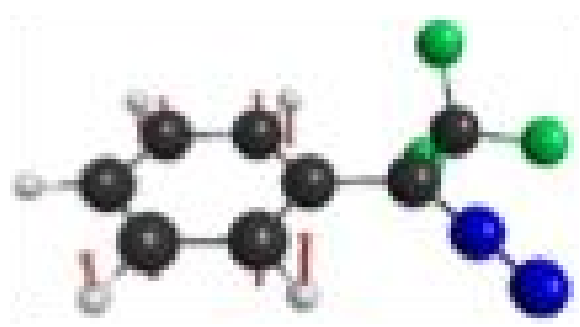

**26**

**Figure S4.** CAM-B3LYP absorption spectrum of the $S_1$ and $S_2$ states of phenyl-trifluoromethyl-diazomethane (**2**). No solvent effect included. (b) Assignment of the bands of calculated spectrum and active mode in band **2**.

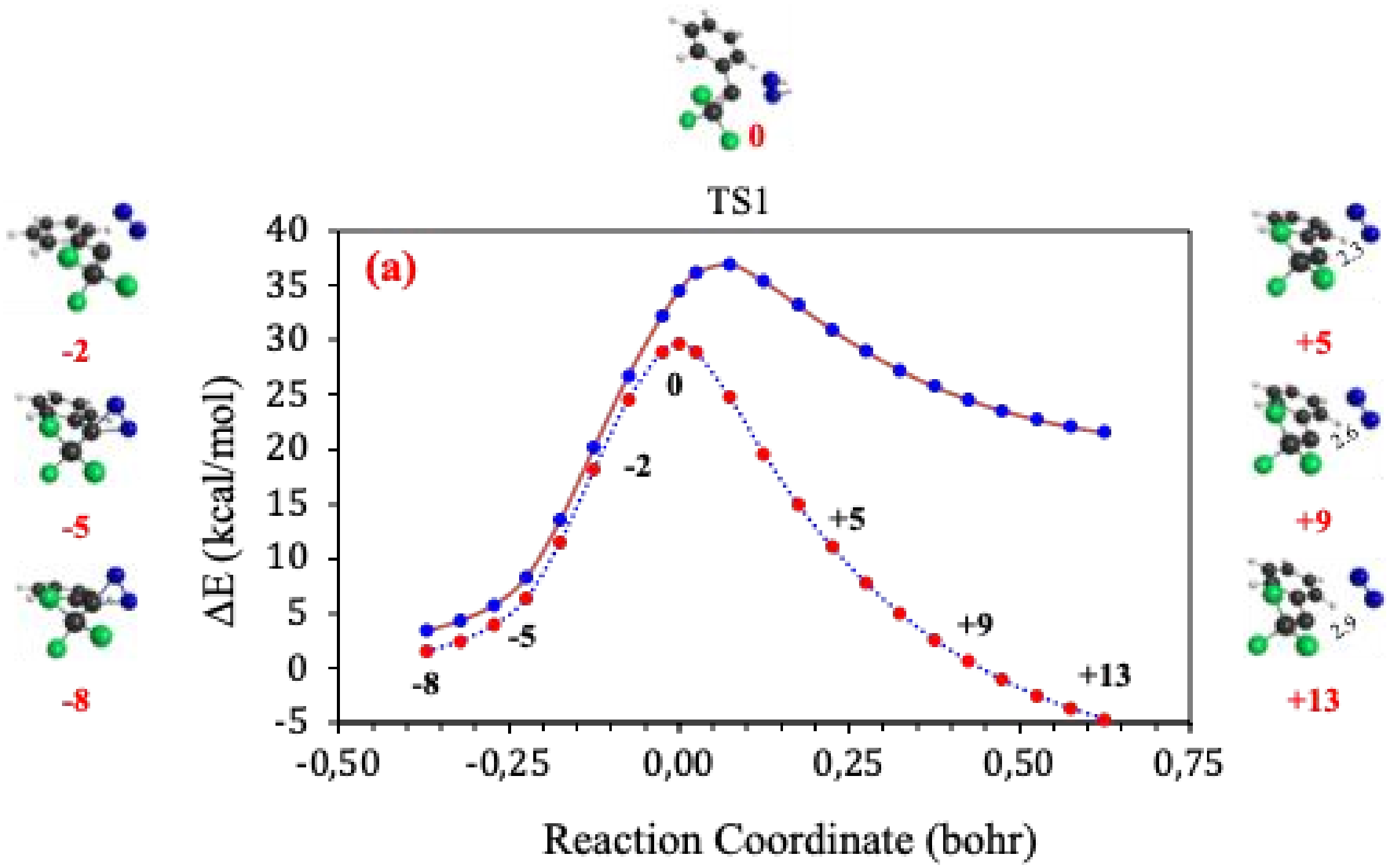


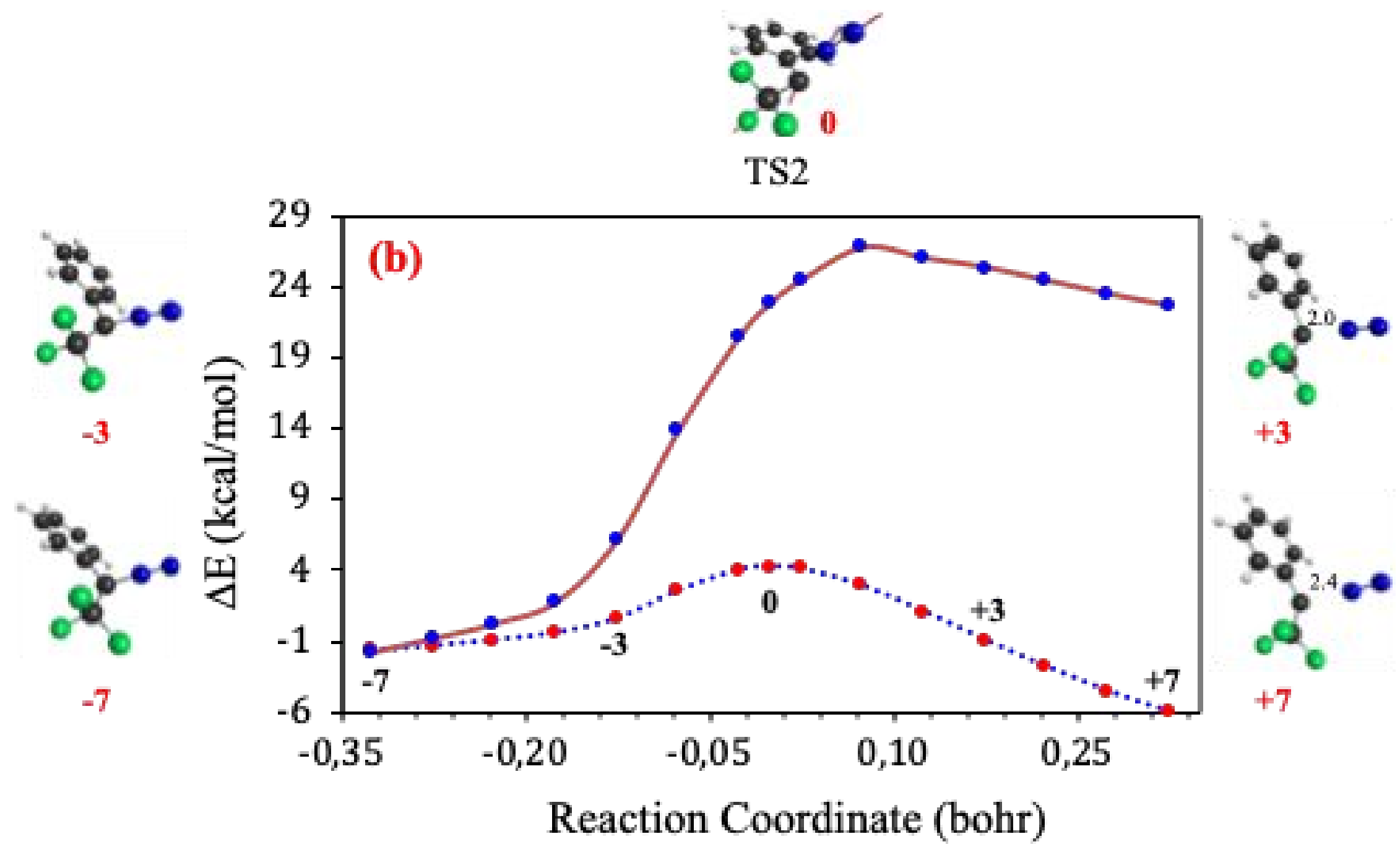


**Figure S5**. (a) CASPT2(red solid line) and CASSCF (blue dotted line) intrinsic reaction coordinate calculations for the dissociation of **1** into $S_0$ carbene and $N_2$ through the transition state TS1. (b) CASPT2(red solid line) and CASSCF (blue dotted line) intrinsic reaction coordinate calculations for the dissociation of **2** into $S_0$ carbene and $N_2$ through the transition state TS2. Energies referred to **1**.

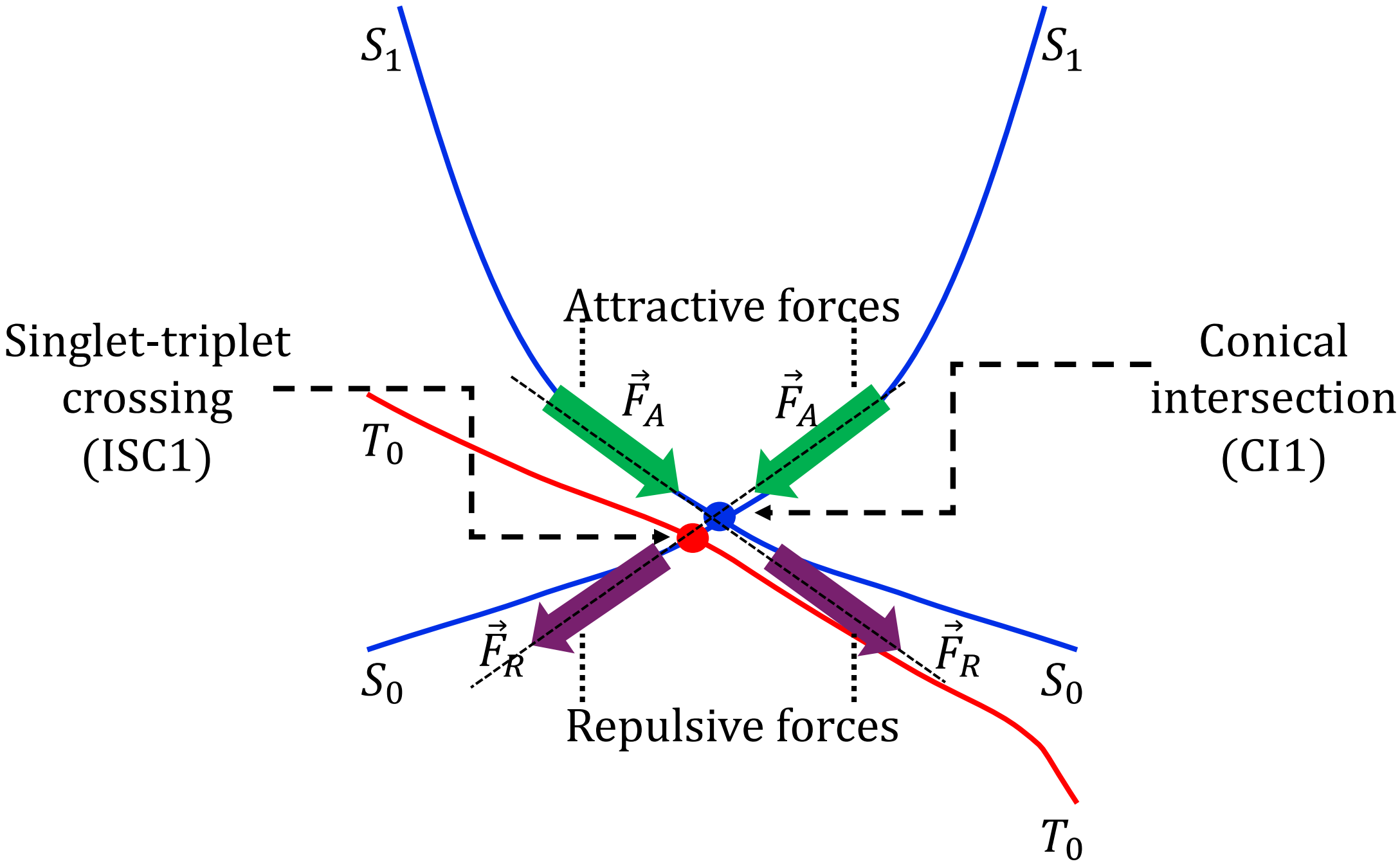


**Figure S6.** Schematic representation between the interaction of the conical intersection **CI1** and the minimum energy singlet-triplet crossing **ISC1**.

# OPTIMIZED GEOMETRIES in Å

**$S_0$ (1). CASPT2 optimized geometry. Reference wave function: CASSCF(14e,14o)/ANO-RCC.**

$E_h$=-716.128 807 44 a.u.

| | | | |
|---|---|---|---|
| 6 | 0.000000 | 0.000000 | 0.000000 |
| 6 | 0.000000 | 0.000000 | 1.477332 |
| 6 | 1.225773 | 0.000000 | 2.151511 |
| 6 | -1.185783 | 0.000000 | 2.218451 |
| 6 | 1.263280 | 0.000000 | 3.541671 |
| 6 | -1.140146 | 0.000000 | 3.607871 |
| 6 | 0.081471 | 0.000000 | 4.274787 |
| 1 | 2.151493 | 0.000000 | 1.595940 |
| 1 | -2.143499 | 0.000000 | 1.723537 |
| 1 | 2.217191 | 0.000000 | 4.048002 |
| 1 | -2.063684 | 0.000000 | 4.167719 |
| 1 | 0.111236 | 0.000000 | 5.354256 |
| 6 | -1.286493 | 0.000000 | -0.779864 |
| 9 | -1.048020 | 0.000000 | -2.092167 |
| 9 | -2.029541 | -1.079882 | -0.499893 |
| 9 | -2.029541 | 1.079882 | -0.499893 |
| 7 | 1.152211 | 0.617655 | -0.695356 |
| 7 | 1.152217 | -0.617637 | -0.695358 |

**$S_0$ (2). CASPT2 optimized geometry. Reference wave function: CASSCF(14e,14o)/ANO-RCC.**

$E_h$=-716.144 652 22 a.u.

| | | | |
|---|---|---|---|
| 6 | 0.000000 | 0.000000 | 0.000000 |
| 6 | 0.000000 | 0.000000 | 1.470226 |
| 6 | 1.202541 | 0.000000 | 2.187821 |
| 6 | -1.201197 | 0.000000 | 2.187065 |
| 6 | 1.202118 | 0.000000 | 3.575828 |
| 6 | -1.194340 | 0.000000 | 3.576717 |
| 6 | 0.003476 | 0.000000 | 4.280282 |
| 1 | 2.143705 | 0.000000 | 1.673417 |
| 1 | -2.140791 | 0.000000 | 1.674999 |
| 1 | 2.137131 | 0.000000 | 4.101362 |
| 1 | -2.128389 | 0.000000 | 4.103948 |
| 1 | 0.004060 | 0.000000 | 5.352452 |
| 6 | -1.212055 | 0.000000 | -0.869774 |
| 9 | -0.874445 | 0.000000 | -2.141695 |
| 9 | -1.978040 | -1.054125 | -0.668368 |
| 9 | -1.978040 | 1.054125 | -0.668368 |
| 7 | 1.134652 | 0.000000 | -0.643372 |
| 7 | 2.137612 | 0.000000 | -1.169993 |

**$S_1$ (1). CASPT2 optimized geometry. Reference wave function: CASSCF(14e,14o)/ANO-RCC.**

$E_h$=- -715.871 865 05 a.u.

| | | | |
|---|---|---|---|
| 6 | 0.000000 | 0.000000 | 0.000000 |
| 6 | 0.000000 | 0.000000 | 1.494991 |
| 6 | 1.210176 | 0.000000 | 2.307343 |
| 6 | -1.288222 | 0.000000 | 2.186113 |
| 6 | 1.116461 | 0.000000 | 3.665288 |
| 6 | -1.330444 | 0.000000 | 3.553659 |
| 6 | -0.147120 | 0.000000 | 4.326940 |
| 1 | 2.166249 | 0.000000 | 1.816675 |
| 1 | -2.201015 | 0.000000 | 1.615792 |
| 1 | 2.025045 | 0.000000 | 4.247298 |
| 1 | -2.292863 | 0.000000 | 4.046144 |
| 1 | -0.199880 | 0.000000 | 5.404057 |
| 6 | -1.254539 | 0.000000 | -0.836497 |
| 9 | -0.936152 | 0.000000 | -2.124973 |
| 9 | -1.999396 | -1.082247 | -0.586144 |
| 9 | -1.999396 | 1.082247 | -0.586144 |
| 7 | 1.081195 | 0.848192 | -0.155074 |
| 7 | 1.081179 | -0.848206 | -0.155077 |

**$S_0$ (1). CASSCF(14e,14o)/ANO-RCC optimized geometry.**

$E_h$=-713.968 316 44 a.u.

| | | | |
|---|---|---|---|
| 6 | 0.000000 | 0.000000 | 0.000000 |
| 6 | 0.000000 | 0.000000 | 1.489776 |
| 6 | 1.219695 | 0.000000 | 2.173105 |
| 6 | -1.184454 | 0.000000 | 2.231207 |
| 6 | 1.252989 | 0.000000 | 3.562192 |
| 6 | -1.145376 | 0.000000 | 3.620289 |
| 6 | 0.070938 | 0.000000 | 4.292080 |
| 1 | 2.144508 | 0.000000 | 1.633572 |
| 1 | -2.136639 | 0.000000 | 1.744776 |
| 1 | 2.199393 | 0.000000 | 4.066444 |
| 1 | -2.065425 | 0.000000 | 4.171193 |
| 1 | 0.096772 | 0.000000 | 5.364104 |
| 6 | -1.288073 | 0.000000 | -0.787979 |
| 9 | -1.053732 | 0.000000 | -2.076444 |
| 9 | -2.018329 | -1.059995 | -0.518449 |
| 9 | -2.018329 | 1.059995 | -0.518449 |
| 7 | 1.159246 | 0.617992 | -0.707877 |
| 7 | 1.159234 | -0.618008 | -0.707879 |

**$S_0$ (2). CASSCF(14e,14o)/ANO-RCC optimized geometry.**

$E_h$=-713.967 219 27 a.u.

| | | | |
|---|---|---|---|
| 6 | 0.000000 | 0.000000 | 0.000000 |
| 6 | 0.000000 | 0.000000 | 1.470226 |
| 6 | 1.202541 | 0.000000 | 2.187821 |
| 6 | -1.201197 | 0.000000 | 2.187065 |
| 6 | 1.202118 | 0.000000 | 3.575828 |
| 6 | -1.194340 | 0.000000 | 3.576717 |
| 6 | 0.003476 | 0.000000 | 4.280282 |
| 1 | 2.143705 | 0.000000 | 1.673417 |
| 1 | -2.140791 | 0.000000 | 1.674999 |
| 1 | 2.137131 | 0.000000 | 4.101362 |
| 1 | -2.128389 | 0.000000 | 4.103948 |
| 1 | 0.004060 | 0.000000 | 5.352452 |
| 6 | -1.212055 | 0.000000 | -0.869774 |
| 9 | -0.874445 | 0.000000 | -2.141695 |
| 9 | -1.978040 | -1.054125 | -0.668368 |
| 9 | -1.978040 | 1.054125 | -0.668368 |
| 7 | 1.134652 | 0.000000 | -0.643372 |
| 7 | 2.137612 | 0.000000 | -1.169993 |

**$S_1$ (1). CASSCF(14e,14o)/ANO-RCC optimized geometry.**

$E_h$=-713.704 119 32 a.u.

| | | | |
|---|---|---|---|
| 6 | 0.000000 | 0.000000 | 0.000000 |
| 6 | 0.000000 | 0.000000 | 1.480208 |
| 6 | 1.296660 | 0.000000 | 2.195378 |
| 6 | -1.231820 | 0.000000 | 2.285179 |
| 6 | 1.338060 | 0.000000 | 3.545355 |
| 6 | -1.167161 | 0.000000 | 3.635240 |
| 6 | 0.115827 | 0.000000 | 4.334333 |
| 1 | 2.202764 | 0.000000 | 1.625615 |
| 1 | -2.183381 | 0.000000 | 1.798793 |
| 1 | 2.283625 | 0.000000 | 4.050197 |
| 1 | -2.071440 | 0.000000 | 4.210806 |
| 1 | 0.153132 | 0.000000 | 5.402423 |
| 6 | -1.297837 | 0.000000 | -0.782034 |
| 9 | -1.068522 | 0.000000 | -2.069630 |
| 9 | -2.015437 | -1.061506 | -0.501104 |
| 9 | -2.015437 | 1.061506 | -0.501104 |
| 7 | 1.058961 | 0.810779 | -0.586384 |
| 7 | 1.058952 | -0.810777 | -0.586389 |

**$S_0$ (1). CAM-B3LYP/def2-TZVPP optimized geometry.**

$E_h$=-716.833 521 39 a.u.

| | | | |
|---|---|---|---|
| 6 | 0.000000 | 0.000000 | 0.000000 |
| 6 | 0.000000 | 0.000000 | 1.481583 |
| 6 | 1.216853 | 0.000000 | 2.158641 |
| 6 | -1.179688 | 0.000041 | 2.221289 |
| 6 | 1.252281 | 0.000040 | 3.541621 |
| 6 | -1.137395 | 0.000081 | 3.603093 |
| 6 | 0.076271 | 0.000081 | 4.269383 |
| 1 | 2.146276 | -0.000031 | 1.609069 |
| 1 | -2.138783 | 0.000042 | 1.727888 |
| 1 | 2.206400 | 0.000040 | 4.049547 |
| 1 | -2.062768 | 0.000113 | 4.161733 |
| 1 | 0.104075 | 0.000112 | 5.349862 |
| 6 | -1.293288 | -0.000293 | -0.775471 |
| 9 | -1.063844 | -0.000393 | -2.088747 |
| 9 | -2.037273 | -1.078980 | -0.494359 |
| 9 | -2.037626 | 1.078216 | -0.494580 |
| 7 | 1.142946 | 0.603984 | -0.698090 |
| 7 | 1.143255 | -0.603398 | -0.698096 |

**$S_0$ (2). CAM-B3LYP/def2-TZVPP optimized geometry.**

$E_h$=-716.854 483 03 a.u.

| | | | |
|---|---|---|---|
| 6 | 0.000000 | 0.000000 | 0.000000 |
| 6 | 0.000000 | 0.000000 | 1.464529 |
| 6 | 1.197817 | 0.000000 | 2.179386 |
| 6 | -1.199594 | 0.000000 | 2.175407 |
| 6 | 1.194392 | 0.000000 | 3.560866 |
| 6 | -1.192926 | 0.000000 | 3.557964 |
| 6 | -0.000444 | 0.000000 | 4.260055 |
| 1 | 2.145373 | 0.000000 | 1.658080 |
| 1 | -2.144525 | 0.000000 | 1.654386 |
| 1 | 2.135427 | 0.000000 | 4.093058 |
| 1 | -2.134645 | 0.000000 | 4.089012 |
| 1 | -0.001614 | 0.000000 | 5.340592 |
| 6 | -1.220245 | -0.000021 | -0.854147 |
| 9 | -0.897464 | -0.000059 | -2.153431 |
| 9 | -1.998977 | -1.073265 | -0.634501 |
| 9 | -1.998974 | 1.073238 | -0.634565 |
| 7 | 1.127414 | 0.000000 | -0.645047 |
| 7 | 2.122159 | -0.000005 | -1.161719 |

**$S_1$ (1). CAM-B3LYP/def2-TZVPP optimized geometry.**

$E_h$=-716.706 774 88 a.u.

| | | | |
|---|---|---|---|
| 6 | 0.000000 | 0.000000 | 0.000000 |
| 6 | 0.000000 | 0.000000 | 1.429866 |
| 6 | 1.237591 | 0.000000 | 2.103796 |
| 6 | -1.185647 | -0.000269 | 2.190318 |
| 6 | 1.283355 | -0.000093 | 3.476398 |
| 6 | -1.126171 | -0.000401 | 3.564285 |
| 6 | 0.103455 | -0.000271 | 4.211129 |
| 1 | 2.144012 | 0.000206 | 1.520595 |
| 1 | -2.146146 | -0.000438 | 1.701566 |
| 1 | 2.237212 | -0.000010 | 3.983551 |
| 1 | -2.039999 | -0.000623 | 4.140428 |
| 1 | 0.142054 | -0.000342 | 5.291550 |
| 6 | -1.299351 | 0.000544 | -0.780076 |
| 9 | -1.109426 | 0.000741 | -2.086330 |
| 9 | -2.039999 | -1.079672 | -0.472483 |
| 9 | -2.039341 | 1.081134 | -0.472177 |
| 7 | 1.175039 | 0.627676 | -0.741142 |
| 7 | 1.174170 | -0.629870 | -0.741099 |

**$S_1/S_0$ CI1 SA4-CASSCF(14e,14o)/ANO-RCC conical intersection.**

$E_h$=-713.900 504 27 au

| | | | |
|---|---|---|---|
| 6 | 0.000000 | 0.000000 | 0.000000 |
| 6 | 0.000000 | 0.000000 | 1.409642 |
| 6 | 1.202957 | 0.000000 | 2.175840 |
| 6 | -1.231440 | 0.000000 | 2.138496 |
| 6 | 1.166836 | 0.000000 | 3.556663 |
| 6 | -1.243037 | -0.000021 | 3.516246 |
| 6 | -0.048274 | 0.000000 | 4.243691 |
| 1 | 2.149122 | 0.000000 | 1.685190 |
| 1 | -2.164576 | 0.000016 | 1.620369 |
| 1 | 2.090222 | -0.000016 | 4.101760 |
| 1 | -2.183616 | -0.000036 | 4.031076 |
| 1 | -0.066166 | 0.000016 | 5.315242 |
| 6 | -1.277891 | 0.000000 | -0.813604 |
| 9 | -1.046333 | -0.000021 | -2.097667 |
| 9 | -2.018622 | -1.059390 | -0.551008 |
| 9 | -2.018601 | 1.059416 | -0.551036 |
| 7 | 1.148632 | 0.000000 | -0.847941 |
| 7 | 2.300683 | 0.000020 | -0.541973 |

**$S_2/S_1$ CI2 SA4-CASSCF(14e,14o)/ANO-RCC conical intersection.**

$E_h$=-713.818 029 89 au

| | | | |
|---|---|---|---|
| 6 | 0.000000 | 0.000000 | 0.000000 |
| 6 | 0.000000 | 0.000000 | 1.392384 |
| 6 | 1.233378 | 0.000000 | 2.131537 |
| 6 | -1.216622 | -0.040062 | 2.156938 |
| 6 | 1.236798 | -0.078811 | 3.504254 |
| 6 | -1.183648 | -0.136943 | 3.527056 |
| 6 | 0.036506 | -0.159962 | 4.226642 |
| 1 | 2.161432 | 0.066501 | 1.603203 |
| 1 | -2.160303 | -0.009557 | 1.656242 |
| 1 | 2.173401 | -0.078265 | 4.026447 |
| 1 | -2.107379 | -0.183136 | 4.069723 |
| 1 | 0.048059 | -0.221355 | 5.295716 |
| 6 | -1.196003 | 0.065213 | -0.897018 |
| 9 | -0.851190 | -0.090685 | -2.153955 |
| 9 | -2.059649 | -0.880902 | -0.601326 |
| 9 | -1.850962 | 1.212875 | -0.821867 |
| 7 | 1.224899 | 0.520544 | -0.676136 |
| 7 | 1.745972 | 1.535448 | -0.935981 |

**$S_2/S_1$ double excitation SA4-CASSCF(14e,14o)/ANO-RCC conical intersection.**

$E_h$=-713.810 541 48

| | | | |
|---|---|---|---|
| 6 | 0.000000 | 0.000000 | 0.000000 |
| 6 | 0.000000 | 0.000000 | 1.424227 |
| 6 | 1.280018 | 0.000000 | 2.150198 |
| 6 | -1.224158 | 0.002158 | 2.223701 |
| 6 | 1.317175 | -0.034357 | 3.503788 |
| 6 | -1.171536 | -0.040463 | 3.577086 |
| 6 | 0.100506 | -0.064178 | 4.282145 |
| 1 | 2.189966 | 0.043860 | 1.589806 |
| 1 | -2.173127 | 0.017604 | 1.732234 |
| 1 | 2.261615 | -0.029497 | 4.010967 |
| 1 | -2.080852 | -0.055198 | 4.144345 |
| 1 | 0.131931 | -0.078410 | 5.350513 |
| 6 | -1.248308 | -0.064831 | -0.830942 |
| 9 | -0.966819 | 0.032491 | -2.107552 |
| 9 | -1.920628 | -1.186079 | -0.663841 |
| 9 | -2.060638 | 0.926115 | -0.530049 |
| 7 | 1.163562 | -0.787258 | -0.728068 |
| 7 | 2.158344 | -0.304750 | -1.110210 |

**$S_3/S_2$ CI3 SA4-CASSCF(14e,14o)/ANO-RCC conical intersection.**

$E_h$=-713.802 589 28

| | | | |
|---|---|---|---|
| 6 | 0.000000 | 0.000000 | 0.000000 |
| 6 | 0.000000 | 0.000000 | 1.404496 |
| 6 | 1.211173 | 0.000000 | 2.205148 |
| 6 | -1.241143 | 0.000000 | 2.164728 |
| 6 | 1.164542 | 0.000021 | 3.611019 |
| 6 | -1.248711 | 0.000021 | 3.568218 |
| 6 | -0.055546 | 0.000043 | 4.315685 |
| 1 | 2.163997 | -0.000016 | 1.730504 |
| 1 | -2.179099 | -0.000016 | 1.657029 |
| 1 | 2.092132 | 0.000020 | 4.147821 |
| 1 | -2.195349 | 0.000020 | 4.070787 |
| 1 | -0.074608 | 0.000075 | 5.385217 |
| 6 | -1.280313 | -0.000022 | -0.797057 |
| 9 | -1.071003 | -0.000019 | -2.084654 |
| 9 | -2.025021 | -1.058777 | -0.525152 |
| 9 | -2.025062 | 1.058704 | -0.525157 |
| 7 | 1.165695 | 0.000041 | -0.879006 |
| 7 | 2.297326 | 0.000047 | -0.489001 |

**$S_0/T_0$ ISC1 CASSCF(14e,14o)/ANO-RCC intersystem crossing.**

$E_h$=-713.919 928 32 au

| | | | |
|---|---|---|---|
| 6 | 0.000000 | 0.000000 | 0.000000 |
| 6 | 0.000000 | 0.000000 | 1.446713 |
| 6 | 1.196943 | 0.000000 | 2.191817 |
| 6 | -1.217615 | 0.000000 | 2.164754 |
| 6 | 1.172528 | 0.000000 | 3.578387 |
| 6 | -1.228087 | 0.000000 | 3.548660 |
| 6 | -0.035240 | 0.000000 | 4.267544 |
| 1 | 2.142821 | 0.000000 | 1.698425 |
| 1 | -2.153064 | 0.000000 | 1.649948 |
| 1 | 2.099847 | 0.000000 | 4.116831 |
| 1 | -2.167926 | 0.000000 | 4.064930 |
| 1 | -0.048350 | 0.000000 | 5.339575 |
| 6 | -1.282462 | 0.000000 | -0.802199 |
| 9 | -1.063009 | 0.000000 | -2.088716 |
| 9 | -2.019301 | -1.059733 | -0.531445 |
| 9 | -2.019301 | 1.059733 | -0.531445 |
| 7 | 1.130858 | 0.000000 | -0.834072 |
| 7 | 2.289190 | 0.000000 | -0.533816 |

**$S_1/S_0$ CAM-B3LYP/def2-TZVPP conical intersection.**

$E_h$=-716.763 851 823 au

| | | | |
|---|---|---|---|
| 6 | 0.000000 | 0.000000 | 0.000000 |
| 6 | 0.000000 | 0.000000 | 1.369850 |
| 6 | 1.212710 | 0.000000 | 2.143591 |
| 6 | -1.231558 | 0.000150 | 2.116437 |
| 6 | 1.174313 | 0.000463 | 3.507004 |
| 6 | -1.237336 | 0.000487 | 3.478478 |
| 6 | -0.039107 | 0.000718 | 4.184725 |
| 1 | 2.152982 | -0.000258 | 1.626070 |
| 1 | -2.172254 | -0.000103 | 1.594512 |
| 1 | 2.100030 | 0.000600 | 4.064294 |
| 1 | -2.177116 | 0.000608 | 4.011230 |
| 1 | -0.052985 | 0.001212 | 5.265607 |
| 6 | -1.306625 | 0.001551 | -0.754900 |
| 9 | -1.150441 | 0.002425 | -2.067232 |
| 9 | -2.064628 | -1.078451 | -0.449297 |
| 9 | -2.062613 | 1.082706 | -0.447755 |
| 7 | 1.131165 | -0.001362 | -0.946696 |
| 7 | 2.251412 | -0.002802 | -0.533402 |

**$S_2/S_1$ CAM-B3LYP/def2-TZVPP conical intersection.**

$E_h$=-716.706 694 86 au

| | | | |
|---|---|---|---|
| 6 | 0.000000 | 0.000000 | 0.000000 |
| 6 | 0.000000 | 0.000000 | 1.405458 |
| 6 | 1.210844 | 0.000000 | 2.138663 |
| 6 | -1.211323 | -0.058947 | 2.141617 |
| 6 | 1.207127 | -0.088087 | 3.510913 |
| 6 | -1.192496 | -0.170497 | 3.509462 |

| | | | |
|---|---|---|---|
| 6 | 0.010870 | -0.183129 | 4.207860 |
| 1 | 2.148618 | 0.074933 | 1.607573 |
| 1 | -2.155119 | -0.037274 | 1.620205 |
| 1 | 2.145480 | -0.082929 | 4.047440 |
| 1 | -2.127792 | -0.234559 | 4.047932 |
| 1 | 0.012472 | -0.254852 | 5.285934 |
| 6 | -1.166158 | 0.167277 | -0.891692 |
| 9 | -0.818449 | 0.089391 | -2.183917 |
| 9 | -2.082384 | -0.786829 | -0.655244 |
| 9 | -1.821634 | 1.351596 | -0.758835 |
| 7 | 1.237502 | 0.699660 | -0.626972 |
| 7 | 1.696211 | 1.713982 | -0.816556 |

**$S_3/S_2$ CAM-B3LYP/def2-TZVPP conical intersection.**

$E_h$=-716.668 112 94 au

| | | | |
|---|---|---|---|
| 6 | 0.000000 | 0.000000 | 0.000000 |
| 6 | 0.000000 | 0.000000 | 1.479592 |
| 6 | 1.209475 | 0.000000 | 2.182995 |
| 6 | -1.164739 | -0.195851 | 2.233164 |
| 6 | 1.261835 | -0.216806 | 3.575029 |
| 6 | -1.119918 | -0.381952 | 3.629486 |
| 6 | 0.095224 | -0.400174 | 4.300586 |
| 1 | 2.120758 | 0.151211 | 1.625267 |
| 1 | -2.116334 | -0.201119 | 1.723854 |
| 1 | 2.220012 | -0.238347 | 4.073415 |
| 1 | -2.041641 | -0.519661 | 4.176034 |
| 1 | 0.130467 | -0.555063 | 5.368182 |
| 6 | -0.847728 | 0.942258 | -0.703649 |
| 9 | -0.725510 | 0.889694 | -2.030454 |
| 9 | -2.219432 | 0.809499 | -0.477053 |
| 9 | -0.603141 | 2.217391 | -0.302975 |
| 7 | -0.009561 | -1.301159 | -0.486961 |
| 7 | 0.225751 | -2.302324 | 0.070329 |

**$S_0$ carbene. CASPT2 optimized geometry. Reference wave function: CASSCF(8e,8o)/ANO-RCC.**

$E_h$=-606.681 308 12 au

| | | | |
|---|---|---|---|
| 6 | 0.000000 | 0.000000 | 0.000000 |
| 6 | 0.000000 | 0.000000 | 1.424637 |
| 6 | 1.291363 | 0.000000 | 2.004541 |
| 6 | -1.124346 | 0.000000 | 2.293626 |
| 6 | 1.464405 | 0.000000 | 3.379315 |
| 6 | -0.948842 | 0.000000 | 3.664128 |
| 6 | 0.341692 | 0.000000 | 4.204588 |
| 1 | 2.131794 | 0.000000 | 1.326375 |
| 1 | -2.120944 | 0.000000 | 1.883634 |
| 1 | 2.454581 | 0.000000 | 3.808479 |
| 1 | -1.806471 | 0.000000 | 4.320074 |
| 1 | 0.467683 | 0.000000 | 5.278350 |
| 6 | -1.376765 | 0.000000 | -0.621622 |
| 9 | -1.328963 | 0.000000 | -1.957253 |
| 9 | -2.088401 | -1.094769 | -0.268940 |
| 9 | -2.088401 | 1.094769 | -0.268940 |

**$S_1$ carbene. CASPT2 optimized geometry. Reference wave function: CASSCF(8e,8o)/ANO-RCC.**

$E_h$=-606.656 442 73 au

| | | | |
|---|---|---|---|
| 6 | 0.000000 | 0.000000 | 0.000000 |
| 6 | 0.000000 | 0.000000 | 1.348206 |
| 6 | 1.228559 | 0.000000 | 2.103598 |
| 6 | -1.249581 | 0.000000 | 2.084192 |
| 6 | 1.198662 | 0.000000 | 3.476561 |
| 6 | -1.237243 | 0.000000 | 3.454956 |
| 6 | -0.023620 | 0.000000 | 4.170133 |
| 1 | 2.163891 | 0.000000 | 1.565200 |
| 1 | -2.180773 | 0.000000 | 1.538335 |
| 1 | 2.127666 | 0.000000 | 4.027811 |
| 1 | -2.174990 | 0.000000 | 3.991823 |
| 1 | -0.033485 | 0.000000 | 5.248870 |
| 6 | -1.000261 | 0.000000 | -1.077220 |
| 9 | -0.423280 | 0.000000 | -2.282857 |
| 9 | -1.807048 | -1.079309 | -1.018114 |
| 9 | -1.807048 | 1.079309 | -1.018114 |

**$T_0$ carbene. CASPT2 optimized geometry. Reference wave function: CASSCF(8e,8o)/ANO-RCC.**

$E_h$=-606.688 277 78 au

| | | | |
|---|---|---|---|
| 6 | 0.000000 | 0.000000 | 0.000000 |
| 6 | 0.000000 | 0.000000 | 1.397761 |
| 6 | 1.221812 | 0.000000 | 2.112280 |
| 6 | -1.216279 | 0.000000 | 2.128358 |
| 6 | 1.223073 | 0.000000 | 3.495916 |
| 6 | -1.194280 | 0.000000 | 3.512871 |
| 6 | 0.018883 | 0.000000 | 4.204316 |
| 1 | 2.150341 | 0.000000 | 1.560764 |
| 1 | -2.156647 | 0.000000 | 1.597862 |
| 1 | 2.162666 | 0.000000 | 4.028938 |
| 1 | -2.126706 | 0.000000 | 4.058615 |
| 1 | 0.026038 | 0.000000 | 5.284109 |
| 6 | -1.073908 | 0.000000 | -1.008455 |
| 9 | -0.571737 | 0.000000 | -2.247245 |
| 9 | -1.873853 | -1.079135 | -0.897195 |
| 9 | -1.873853 | 1.079135 | -0.897195 |

**$S_0$ carbene. CAM-B3LYP/def2-TZVPP.**

$E_h$=-607.281 057 46 au

| | | | |
|---|---|---|---|
| 6 | 0.000000 | 0.000000 | 0.000000 |
| 6 | 0.000000 | 0.000000 | 1.415315 |
| 6 | 1.279900 | 0.000000 | 2.010344 |
| 6 | -1.129166 | 0.001892 | 2.271976 |
| 6 | 1.435706 | -0.000174 | 3.379551 |
| 6 | -0.970288 | 0.003741 | 3.635983 |
| 6 | 0.308799 | 0.002057 | 4.186246 |
| 1 | 2.130370 | -0.000177 | 1.344471 |
| 1 | -2.121976 | 0.002845 | 1.850417 |
| 1 | 2.421548 | -0.001064 | 3.821505 |
| 1 | -1.834394 | 0.006077 | 4.284915 |
| 1 | 0.422840 | 0.002862 | 5.262265 |
| 6 | -1.345281 | -0.006222 | -0.658426 |
| 9 | -1.266457 | -0.024163 | -1.991909 |
| 9 | -2.077770 | -1.091218 | -0.314437 |
| 9 | -2.060647 | 1.097393 | -0.339728 |

**$S_1$ carbene. CAM-B3LYP/def2-TZVPP.**

$E_h$=-607.252 381 541 au

| | | | |
|---|---|---|---|
| 6 | 0.000000 | 0.000000 | 0.000000 |
| 6 | 0.000000 | 0.000000 | 1.357119 |
| 6 | 1.226842 | 0.000000 | 2.080302 |
| 6 | -1.216811 | 0.005076 | 2.111094 |
| 6 | 1.229728 | 0.007391 | 3.453388 |
| 6 | -1.177283 | 0.007341 | 3.480199 |
| 6 | 0.036445 | 0.010506 | 4.163649 |
| 1 | 2.152444 | -0.001050 | 1.525854 |
| 1 | -2.158887 | 0.011183 | 1.586658 |
| 1 | 2.172258 | 0.010355 | 3.982739 |
| 1 | -2.105619 | 0.012300 | 4.034479 |
| 1 | 0.048383 | 0.014639 | 5.243901 |
| 6 | -0.853082 | 0.004273 | -1.173755 |
| 9 | -0.692670 | -1.100662 | -1.923306 |
| 9 | -2.166206 | 0.062052 | -0.845077 |
| 9 | -0.609525 | 1.051819 | -1.978313 |

**$T_0$ carbene. CAM-B3LYP/def2-TZVPP.**

$E_h$=-607.290 085 64 au

| | | | |
|---|---|---|---|
| 6 | 0.000000 | 0.000000 | 0.000000 |
| 6 | 0.000000 | 0.000000 | 1.386705 |
| 6 | 1.218471 | 0.000000 | 2.104436 |
| 6 | -1.211091 | -0.000698 | 2.123136 |
| 6 | 1.220027 | -0.000558 | 3.479343 |
| 6 | -1.186283 | -0.000855 | 3.498473 |
| 6 | 0.022172 | -0.000984 | 4.185678 |
| 1 | 2.148093 | 0.000660 | 1.553842 |
| 1 | -2.153845 | -0.002877 | 1.595858 |
| 1 | 2.160412 | -0.000484 | 4.012547 |
| 1 | -2.118079 | -0.001501 | 4.046756 |
| 1 | 0.030353 | -0.001645 | 5.266291 |
| 6 | -1.013281 | 0.018554 | -1.057742 |

| | | | |
|---|---|---|---|
| 9 | -0.455824 | -0.090931 | -2.268537 |
| 9 | -1.899930 | -0.991370 | -0.933546 |
| 9 | -1.737773 | 1.156011 | -1.054258 |

**$S_0/T_0$ ISC2 CASSCF(8e,8o)/ANO-RCC intersystem crossing.**

$E_h$=-604.797 661 25 au

| | | | |
|---|---|---|---|
| 6 | 0.000000 | 0.000000 | 0.000000 |
| 6 | 0.000000 | 0.000000 | 1.447289 |
| 6 | 1.282719 | 0.000000 | 2.021213 |
| 6 | -1.120070 | 0.000000 | 2.310233 |
| 6 | 1.455636 | 0.000000 | 3.399423 |
| 6 | -0.945292 | 0.000000 | 3.681375 |
| 6 | 0.340570 | 0.000000 | 4.226781 |
| 1 | 2.127110 | 0.000000 | 1.362581 |
| 1 | -2.111967 | 0.000000 | 1.912371 |
| 1 | 2.441651 | 0.000000 | 3.820050 |
| 1 | -1.799946 | 0.000000 | 4.328766 |
| 1 | 0.464937 | 0.000000 | 5.292403 |
| 6 | -1.375225 | 0.000000 | -0.647348 |
| 9 | -1.286917 | 0.000000 | -1.955747 |
| 9 | -2.073485 | -1.071971 | -0.316693 |
| 9 | -2.073485 | 1.071971 | -0.316693 |

**$S_0/T_0$ ISC2 CAM-B3LYP/def2-TZVPP intersystem crossing.**

$E_h$=-607.280 710 93 au

| | | | |
|---|---|---|---|
| 6 | 0.000000 | 0.000000 | 0.000000 |
| 6 | 0.000000 | 0.000000 | 1.410207 |
| 6 | 1.269324 | 0.000000 | 2.028385 |
| 6 | -1.144921 | -0.000020 | 2.244600 |
| 6 | 1.396521 | -0.000019 | 3.399760 |
| 6 | -1.011605 | -0.000018 | 3.612378 |
| 6 | 0.255263 | -0.000038 | 4.188076 |
| 1 | 2.136014 | 0.000032 | 1.383485 |
| 1 | -2.129098 | -0.000037 | 1.802584 |
| 1 | 2.374390 | -0.000019 | 3.859384 |
| 1 | -1.889313 | -0.000017 | 4.242943 |
| 1 | 0.349827 | -0.000069 | 5.265789 |
| 6 | -1.306236 | -0.000023 | -0.718082 |
| 9 | -1.164713 | -0.000192 | -2.046651 |
| 9 | -2.046186 | -1.091852 | -0.417145 |
| 9 | -2.046049 | 1.091963 | -0.417390 |

**$S_1/S_0$ CI4 CASSCF(8e,8o)/ANO-RCC.**

$E_h$=-604.766 947 56 au

| | | | |
|---|---|---|---|
| 6 | 0.000000 | 0.000000 | 0.000000 |
| 6 | 0.000000 | 0.000000 | 1.352734 |
| 6 | 1.254393 | 0.000000 | 2.081554 |
| 6 | -1.224394 | 0.001175 | 2.138930 |
| 6 | 1.271259 | 0.000308 | 3.450492 |
| 6 | -1.171328 | 0.001956 | 3.505551 |
| 6 | 0.067480 | 0.001522 | 4.186646 |
| 1 | 2.165352 | -0.000808 | 1.518444 |
| 1 | -2.168300 | 0.001242 | 1.633801 |
| 1 | 2.209102 | 0.000091 | 3.970730 |
| 1 | -2.083176 | 0.003027 | 4.070286 |
| 1 | 0.091043 | 0.002016 | 5.257514 |
| 6 | -1.077480 | 0.000207 | -1.021036 |
| 9 | -0.585950 | -0.029167 | -2.236974 |
| 9 | -1.875408 | -1.044891 | -0.898816 |
| 9 | -1.838799 | 1.075429 | -0.934745 |

**$S_1/S_0$ CI4 CAM-B3LYP/def2-TZVPP.**

$E_h$=-607.241 539 82 au

| | | | |
|---|---|---|---|
| 6 | 0.000000 | 0.000000 | 0.000000 |
| 6 | 0.000000 | 0.000000 | 1.347457 |
| 6 | 1.237357 | 0.000000 | 2.068071 |
| 6 | -1.209547 | 0.003589 | 2.120241 |
| 6 | 1.251699 | 0.001004 | 3.433319 |
| 6 | -1.156211 | 0.006376 | 3.484980 |
| 6 | 0.062921 | 0.004203 | 4.156323 |

| | | | |
|---|---|---|---|
| 1 | 2.158646 | -0.001825 | 1.505309 |
| 1 | -2.157649 | 0.002909 | 1.606745 |
| 1 | 2.198007 | 0.000080 | 3.955937 |
| 1 | -2.077983 | 0.009463 | 4.050115 |
| 1 | 0.085854 | 0.005487 | 5.236117 |
| 6 | -0.885475 | 0.004652 | -1.150934 |
| 9 | -0.537516 | -0.920069 | -2.062958 |
| 9 | -2.166763 | -0.245717 | -0.801302 |
| 9 | -0.891697 | 1.185804 | -1.803396 |
